# Wake-Promoting and EEG Spectral Effects of Modafinil After Acute or Chronic Administration in the R6/2 Mouse Model of Huntington’s Disease

**DOI:** 10.1007/s13311-020-00849-y

**Published:** 2020-04-15

**Authors:** Szilvia Vas, Jackie M. Casey, Will T. Schneider, Lajos Kalmar, A. Jennifer Morton

**Affiliations:** 1 Department of Physiology, Development and Neuroscience, University of Cambridge, Downing Street, Cambridge, CB2 3DY UK; 2 Department of Veterinary Medicine, University of Cambridge, Madingley Road, Cambridge, CB3 0ES UK

**Keywords:** Gamma oscillation, theta, delta, EEG power, rapid eye movement sleep, cognitive enhancer

## Abstract

Huntington’s disease (HD) is characterised by progressive symptoms including cognitive deficits and sleep/wake disturbances reflected in an abnormal electroencephalography (EEG). Modafinil, a wake-promoting and cognitive-enhancing drug, has been considered as a treatment for HD. We used HD (R6/2) mice to investigate the potential for using modafinil to treat sleep-wake disturbance in HD. R6/2 mice show sleep-wake and EEG changes similar to those seen in HD patients, with increased rapid eye movement sleep (REMS), decreased wakefulness/increased non-REMS (NREMS), and pathological changes in EEG spectra, particularly an increase in gamma power. We recorded EEG from R6/2 and wild-type mice treated with modafinil acutely (with single doses between 25 and 100 mg/kg; at 12 and 16 weeks of age), or chronically (64 mg/kg modafinil/day from 6 to 15 weeks). Acutely, modafinil increased wakefulness in R6/2 mice and restored NREMS to wild-type levels at 12 weeks. It also suppressed the pathologically increased REMS. This was accompanied by decreased delta power, increased peak frequency of theta, and increased gamma power. At 16 weeks, acute modafinil also restored wakefulness and NREMS to wild-type levels. However, whilst REMS decreased, it did not return to normal levels. By contrast, in the chronic treatment group, modafinil-induced wakefulness was maintained at 15 weeks (after 9 weeks of treatment). Interestingly, chronic modafinil also caused widespread suppression of power across the EEG spectra, including a reduction in gamma that increases pathologically in R6/2 mice. The complex EEG effects of modafinil in R6/2 mice should provide a baseline for further studies to investigate the translatability of these result to clinical practice.

**Electronic supplementary material:**

The online version of this article (10.1007/s13311-020-00849-y) contains supplementary material, which is available to authorized users.

## Introduction

Huntington’s disease (HD) is a complex progressive genetic neurodegenerative disorder characterised by motor, cognitive, and affective symptoms [1]. Disturbances of sleep-wake behaviour, including sleep fragmentation [2, 3], abnormality of rapid eye movement sleep (REMS) [4, 5], and daytime sleepiness [6, 7] are widespread problems in HD patients that frequently appear early in the course of the disease [8, 9]. Pathological EEG spectral abnormalities are also seen in HD patients [3, 9, 10].

Alterations of the sleep-wake cycle and electroencephalogram (EEG) found in HD patients are recapitulated in a number of HD mouse models, including the transgenic lines of R6/2 [11, 12], R6/1 [13, 14], and Q175 [15–17] mice. We used the R6/2 line for our studies [18]. These mice show a phenotype mirroring important symptoms seen in HD patients, including deteriorating motor and cognitive functions [19, 20]. Previous studies show that R6/2 mice have fragmented sleep, accompanied by abnormal REMS across the day/night [11, 12]. R6/2 mice also show progressive changes in quantitative EEG (qEEG), in particular a pathological increase in gamma oscillation power [11, 12].

It has been suggested that disruption of sleep contributes to cognitive decline in HD [21–23]. Thus, treatments aimed at improving the sleep-wake pattern in HD patients may also have a beneficial knock-on effect on cognitive symptoms of the disease. We showed previously using a combination of modafinil (Provigil) and alprazolam that treating sleep-wake deficits in R6/2 mice has a beneficial effect on cognitive performance and arousability [9, 21]. To date, however, only one study has examined the effect of modafinil on mood and cognition in HD patients [24]. In that study, although modafinil increased alertness, it did not improve cognitive function. However, the effect of only a single dose of modafinil was used. Given that HD patients may respond to drugs differently from normal subjects, and that modafinil is used as a cognitive enhancer in both normal and patient groups (for references, see [25]), modafinil remains an interesting candidate for treating sleep-wake disorder in HD [26].

Modafinil is a wake-promoting agent most widely used for treating excessive daytime sleepiness in several neuropsychiatric conditions including narcolepsy [27] and Parkinson’s disease [28]. Modafinil evokes its robust wake-promoting effect in the brain by increasing the level of dopamine, norepinephrine, glutamate, serotonin, histamine, and hypocretin/orexin whilst decreasing the release of GABA [29]. It is also a putative cognitive enhancer although in this respect the literature is controversial [25, 30]. It improves memory [31], executive function [32], and increases alertness and response accuracy in healthy subjects [25]. Importantly, the cognitive enhancer effect of modafinil has also been reported in a number of different neurological conditions including schizophrenia [33, 34] and attention deficit hyperactivity disorder [35]. Modafinil has also been shown to be effective when given as an adjunct to standard treatment for treating mood disorders [36, 37]. This is particularly relevant in HD, since low mood and depression occur in a considerable number of patients [38]. Moreover, pre-symptomatic HD gene–positive individuals with subjective sleep problems have shown significantly poorer neuropsychiatric outcomes compared to those not reporting sleep problems [23]. Thus, targeting sleep-wake disorder with a drug that may also improve cognitive function would potentially be doubly beneficial.

Here we investigate the acute effect of modafinil on sleep and wakefulness at different stages of disease in R6/2 mice. We also administered modafinil chronically for 9 weeks to determine if there was any long-term effect of modafinil on the progression of sleep-wake abnormalities in HD mice. We found that acutely modafinil was wake-promoting in R6/2 mice and reduced the pathologically increased REMS, although the effect waned as the disease progressed. Interestingly, when given chronically, modafinil maintained its wake-promoting effect to end stage, and reduced the pathologically increased gamma oscillations, an effect that persisted after washout.

## Materials and Methods

### Ethical Statement

All experiments were conducted under the authority of the United Kingdom Animals (Scientific Procedures) Act 1986 Amendment Regulations 2012, and with the approval of the University of Cambridge Animal Welfare and Ethical Review Body.

### Animal Numbers and Husbandry

Male R6/2 mice and their wild-type (WT) littermates (C57/BL6J) were taken from a colony established at the University of Cambridge. Tail snips were taken at 3 weeks of age for genotyping and CAG repeat sizing (Laragen, Los Angeles, CA). CAG repeat lengths were measured by GeneMapper software (Life Technologies, NY). R6/2 mice had a mean CAG repeat length of 250 ± 1 (*n* = 52).

Two experiments were conducted. (For an overview of the timeline and drug administration protocol, see Fig. S1 and below.) In the acute experiment, single doses of modafinil were given to 10 R6/2 and 11 WT mice. One R6/2 mouse died from its disease before completion of the late-stage (15–17 weeks) treatments, one WT mouse lost its EEG/EMG implant and was euthanized before completing the study, and one WT mouse died unexpectedly of unknown causes. Due to the cross-over design of the study, we excluded all recordings of these mice from the data analysis. In total, 9/11 WT and 9/10 R6/2 mice completed all cross-over treatments and EEG/EMG recordings. In the chronic experiment, modafinil was administered to 20 R6/2 and 11 WT mice. During the study, one R6/2 mouse died from its disease, and one R6/2 mouse lost its EEG/EMG implant and was euthanized before completing the study. Thus, 18/20 R6/2 and 11/11 WT mice completed the chronic modafinil experiment and were used for the analysis.

After the EEG/EMG implant surgery, animals were housed individually in cages within sound-attenuating chambers. They were kept under a 12:12-h light–dark cycle (30 lx daylight-type fluorescent tubes with lights on at 23:00), at constant temperature (22 ± 1 °C) and humidity (55 ± 10%). Food and water were available ad libitum. R6/2 mice also received a single portion of soft mash each morning (made by soaking chow pellets in hot water), with an additional portion added later in the day for mice aged > 12 weeks. Bedding was changed as needed, approximately once a week for WT mice and twice a week for R6/2 mice.

### EEG/EMG Implantation Surgery

All mice were implanted with EEG and EMG electrodes under isoflurane anaesthesia (1.5–2% in 2 l/m oxygen) as described previously [11]. Briefly, gold-plated stainless steel screw electrodes were placed epidurally above both left and right hemispheres positioned over the frontal (1.5 mm lateral and 1.0 mm anterior to bregma) and parietal (1.5 mm lateral and 1.0 mm anterior to lambda) cortex, for fronto-parietal EEG recordings. EMG signals were acquired from the neck extensor muscles via a pair of stainless steel spring wires (Plastics One Inc., Roanoke, VA). After an 8–10-day recovery period, the mice were connected to the recording cables, and remained connected to the cables throughout the study. The recording cables allowed free movement within the cage.

### EEG/EMG Recordings

Following a 12-day acclimatisation period, we began the treatment program. We recorded EEG and EMG signals (Pinnacle Technology, Lawrence, KS) for 24 h after each treatment started at 11 am (at light off). The EEG/EMG signals were amplified and filtered (EEG, 0.5–100 Hz; EMG, 35 Hz high path filter [HPF]) by head-mounted preamplifiers and amplifiers (8202-DSL and 8206-SL, respectively; Pinnacle Technology, Lawrence, KS). EEG/EMG data were recorded using Vital Recorder software (Kissei Comtec, Matsumoto, Japan) after analogue-to-digital conversion.

### Drug Administration

Modafinil ([(diphenyl-methyl-methyl) sulfinyl]-2 acetamide) was dissolved in 15 *w*/*v* % hydroxypropyl-β-cyclodextrin (β-HPCD) in (0.9%) saline with sonication and gentle warming 1 h before the experiment (vehicle). In the acute experiment, mice were treated with one of three doses (25, 50, and 100 mg/kg) of modafinil, or vehicle given via intraperitoneal (i.p.) injections (1 ml/100 g body weight) at the beginning of the dark (active) period. Doses were chosen based on previous studies [21, 39, 40]. The treatments were performed in a cross-over design (Fig. S1a, c) in which each mouse received all doses of modafinil in a randomized order, with a 3-day washout period between treatments. To compare the effect of acute administration of modafinil at different stages of the disease, mice were treated at both 11–13 weeks of age (early symptomatic stage for R6/2 mice) and at 15–17 weeks of age (mid-late stage). Mice aged 11–13 weeks and 15–17 weeks are henceforth referred as ‘12 weeks’ or ‘16 weeks’, respectively. In the chronic treatment experiment, mice were treated orally in the form of edible pills which contained 64 mg/kg of modafinil in dough, prepared using wheat, sugar, water, oil, and a small quantity of non-toxic blue food dye to ensure an equal distribution of the drug [41], or the equivalent amount of dough. Mice were treated for 9 weeks starting at 6 weeks of age (Fig. S1b). Mice were randomly assigned to treatment groups. In the R6/2 group, 10 mice were treated with modafinil-containing pills and 8 mice were given pills containing vehicle. We deliberately included more mice in the modafinil-treatment group as we expected that some R6/2 mice might stop eating the modafinil-containing pills due from the bitter taste of the drug. In fact, it was the WT mice that stopped eating the modafinil-containing pills (between the 8th and 39th days of the treatment period). For this reason, we did not analyse EEG from the WT mice in the chronic treatment. By contrast, all R6/2 mice ate the pills daily until the end of the study, and data from all R6/2 mice are therefore included.

### Data Analysis

EEG and EMG signals were digitalized using a 256-Hz sampling rate and digitally filtered (EEG, 0.5–100Hz; EMG, 35Hz HPF). Automatic scoring of the EEG traces was performed using SleepSign software (Kissei Comtec, Matsumoto, Japan). For 10s epochs of the EEG traces, we distinguished the following vigilance stages: wake, NREMS, and REMS. The automatic scoring was followed by visual assessment from experienced scorer, blind to treatment, and genotype. We performed quantitative EEG (qEEG) analysis using fast Fourier transformations (FFTs) for consecutive 10s epochs in the frequency range 0.5–90 Hz (frequency resolution of 0.5 Hz, Hanning window). Epochs with movement-induced and other artefacts were discarded. To exclude the interference noise from the electrical network, values at 49- and 50-Hz bins were excluded from the qEEG analysis. The EEG spectral analysis was performed in a state-specific manner by averaging FFT data for each vigilance stage (wakefulness, NREMS, REMS) for the given time period.

### Statistics

To evaluate the effect of single doses of modafinil on the amount of wakefulness, NREMS and REMS for each hour a two-way double repeated measures ANOVA was used (hours and doses, both as repeated variables). For the analysis of different single doses of modafinil on the summarized vigilance data (1–12 h), we used two-way repeated measures ANOVA with the factors: groups (WT and R6/2 mice aged 12 weeks), age of R6/2 mice (12 and 16 weeks, repeated), and doses (repeated). The effect of chronic modafinil treatment on vigilance was analysed at 4 time points (after 6 or 9 weeks of treatment, and after 1 or 2 weeks of washout) by two-way repeated measures ANOVA (hours [1–6], repeated). The effect of modafinil vs. vehicle on the sleep-wake pattern (number and duration of bouts) of wakefulness, NREMS, and REMS was quantified using *t* tests. A bout was defined as an episode without interruptions of more than 1 epoch of any other vigilance stage.

Statistical analyses on qEEG data were performed on raw EEG power data averaged for the first 2 h of both active and passive phases, and binned into standard frequency bands: delta (1–4 Hz), theta (5–9 Hz), alpha (10–14 Hz), and beta (15–30 Hz) as well as low- (31–60 Hz) and high-frequency gamma (61–90 Hz). In all cases, two-way repeated measures ANOVA was used. In the acute study, we evaluated the qEEG alterations of R6/2 mice vs. WT mice (genotype and frequency, repeated) and the effect of modafinil vs. vehicle (frequency and drug, repeated) in each group. In the chronic experiment, the qEEG effect of modafinil was quantified at each of 4 time points (after 6 and 9 weeks of treatment, and then 1 and 2 weeks after washout) with factors being drug and frequency (repeated). For multiple comparisons, Bonferroni post hoc test was used. The interaction effect of ANOVA (for a frequency range) was considered significant only if at least 2 x 1Hz frequency bins showed significant difference compared to the vehicle group. Results were considered statistically significant at *p* < 0.05. All data are shown as mean ± SEM.

## Results

### Acute Modafinil Has a Similar Wake-Promoting Effect in Wild-Type and R6/2 Mice at 12 Weeks of Age

**Fig. 1:**
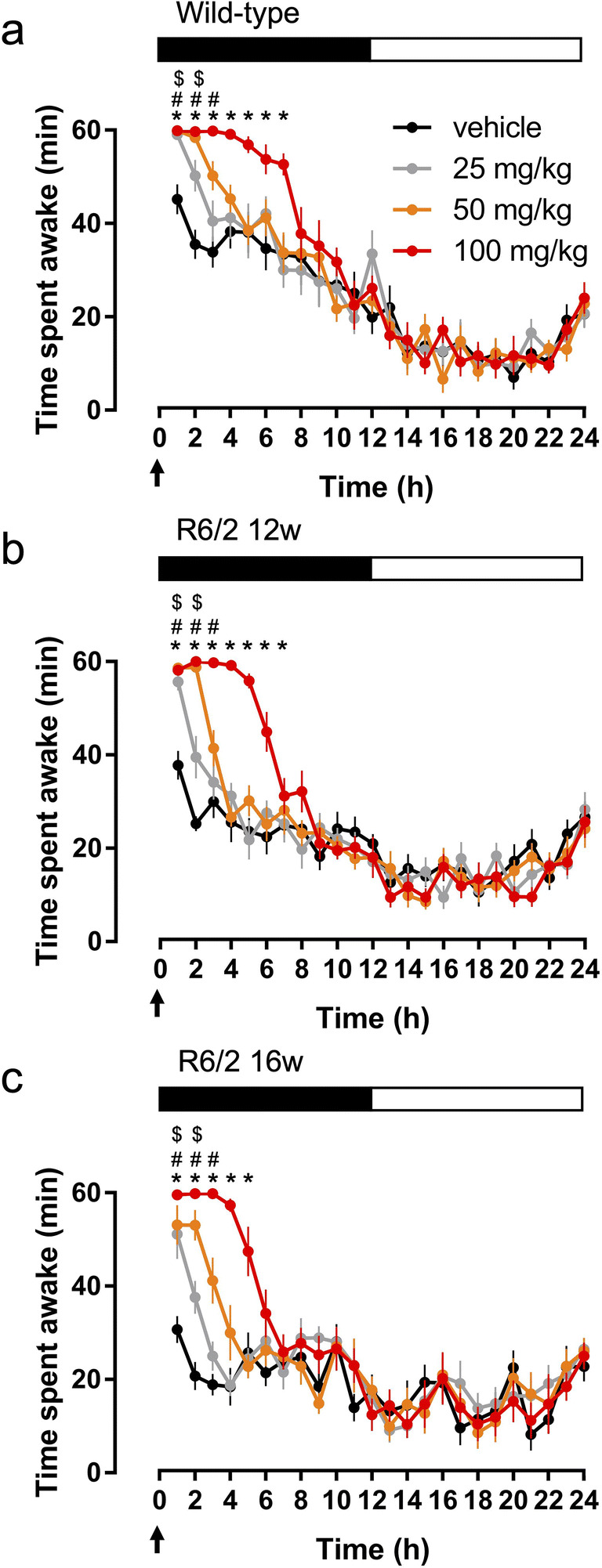
The wake-promoting effect of modafinil in R6/2 and wild-type mice. The hourly amount of the time spent awake across 24 h after treatment with single doses of modafinil (25, 50, or 100 mg/kg; i.p., arrows) is shown compared to vehicle at the beginning of active (dark) phase in wild-type (a) and R6/2 mice aged 12 weeks (b) and 16 weeks (c). The black bar shows the dark period. $, #, and *p < 0.05, compared to the relevant vehicle in case of the 25, 50, and 100 mg/kg modafinil-treated groups, respectively. Data are presented as mean ± standard error of mean (SEM)

Modafinil, given at the beginning of active phase, induced a dose-dependent increase in the time spent awake in WT mice (Fig. 1a, drug: *F*_(3,24)_ = 26.16, *p* < 0.0001, drug × time interact.: *F*_(69,552)_ = 2. 32, *p* < 0.0001). Modafinil induced wakefulness also in R6/2 mice at both 12weeks (Fig. 1b, drug: *F*_(3,24)_ = 37.68, *p* < 0.0001, drug × time interact.: *F*_(69,552)_ = 5.72, *p* < 0.0001) and 16 weeks (Fig. 1c, drug: *F*_(3,24)_ = 16.61, *p* < 0.0001, drug × time interact.: *F*_(69,552)_ = 2.97, *p* < 0.0001). At the 100 mg/kg dose, the wake-promoting effect was similar in WT and R6/2 mice aged 12 weeks, lasting for ~ 7 h. Modafinil was less effective in R6/2 mice aged 16 weeks, lasting for ~ 5 h. At lower doses, modafinil increased the amount of wakefulness by 2 h (for 25 mg/kg) or 3 h (for 50 mg/kg), in both WT mice and R6/2 mice at 12 and 16weeks (Fig. 1a–c).

**Fig. 2:**
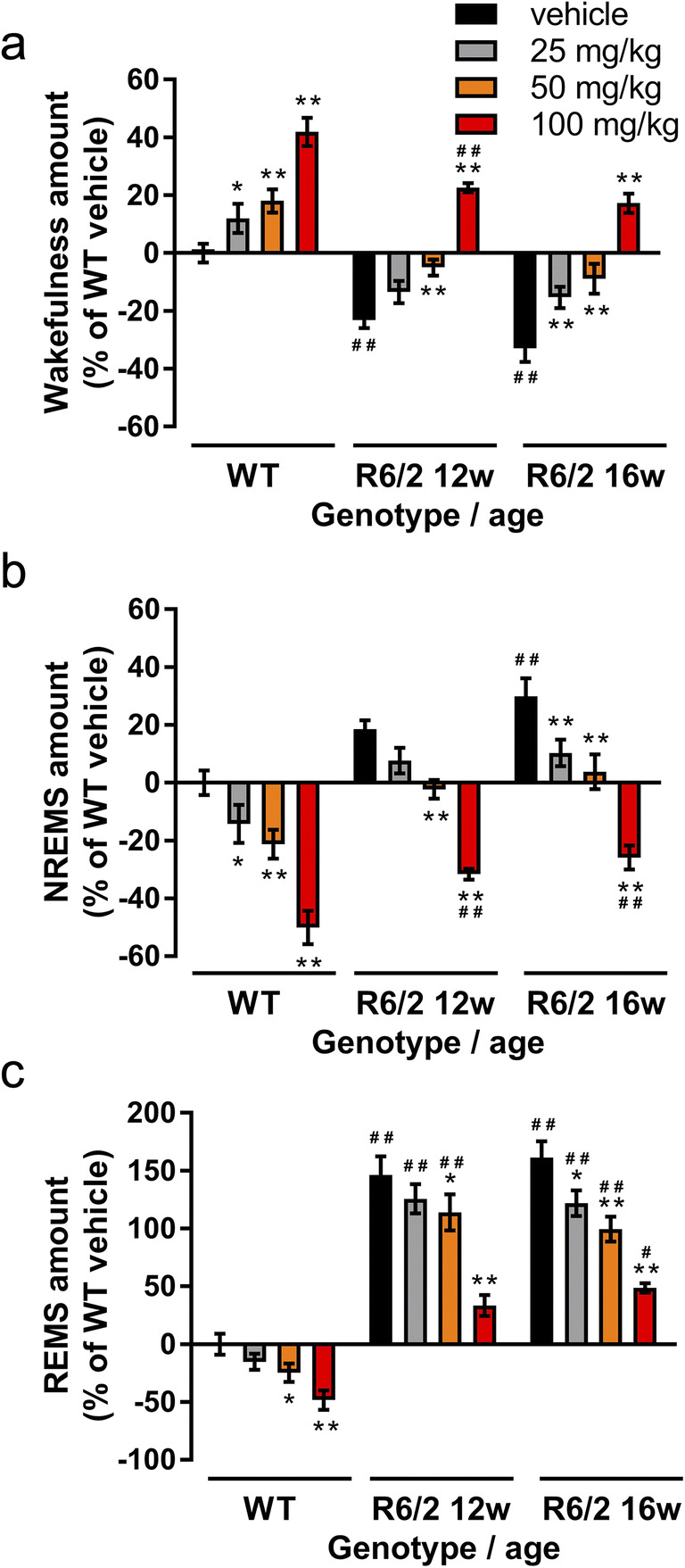
The effect of acute modafinil on the amount of sleep-wake stages in active phase in R6/2 and wild-type mice. Modafinil (25, 50, and 100 mg/kg; i.p.) was administered at the beginning of active phase (11 am) and the time spent awake (a), in non-rapid eye movement sleep (NREMS, b), and in rapid eye movement sleep (REMS, c) was measured in wild-type (WT), and in R6/2 mice aged 12 weeks and 16 weeks. Data are presented as (mean ± SEM) percent changes in the time spent in each vigilance stage relative to the WT vehicle group. *p < 0.05 and **p < 0.01 compared to the relevant vehicle treatment; ^#^*p* < 0.05 and ^##^*p* < 0.01 compared to the WT vehicle group

Analysis of the whole active phase showed that vehicle-treated R6/2 mice at 12 and 16 weeks spent ~ 23% and ~ 33% less time awake respectively than WT mice (group: *F*_(3,96)_ = 58.33, *p* < 0.0001, Fig. 2a). Lower doses of modafinil (25 or 50 mg/kg) restored the amount of wakefulness in R6/2 mice at 12 and 16 weeks to the WT level, whilst 100 mg/kg caused a significant increase in wakefulness in 12-week-old R6/2 mice compared to the WT vehicle-treated mice (Fig. 2a). In parallel with the wake-promoting effect, modafinil dose-dependently reduced NREMS in all groups (Fig. 2b, *F*_(3,96)_ = 62.59, *p* < 0.0001), and restored NREMS amount to the WT level in both 12- and 16-week-old R6/2 mice by both the 25 mg/kg and 50 mg/kg doses, whilst 100 mg/kg caused a further decrease. Although modafinil reduced the pathologically increased REMS amount in R6/2 mice at both 12 and 16 weeks (Fig. 2c, drug: *F*_(3,96)_ = 36.30, *p* < 0.0001, drug × group interact.: *F*_(6,96)_ = 2.51, *p* = 0.0269), only the 100 mg/kg dose given to R6/2 mice aged 12 weeks restored REMS to the WT level.

In the passive phase, modafinil (100 mg/kg) induced a sleep-consolidating effect in 12-week-old R6/2 mice by increasing both the total time and the mean duration of episodes in NREMS. This effect was not seen in WT or 16-week-old R6/2 mice. Modafinil also had no effect on wakefulness, or the amount and mean duration of REMS episodes during the passive phase (Table S1).

### Acute Modafinil Reduces Power of Slow, but Increases Power of High-Frequency Oscillations During Wakefulness in R6/2 and WT Mice

**Fig. 3:**
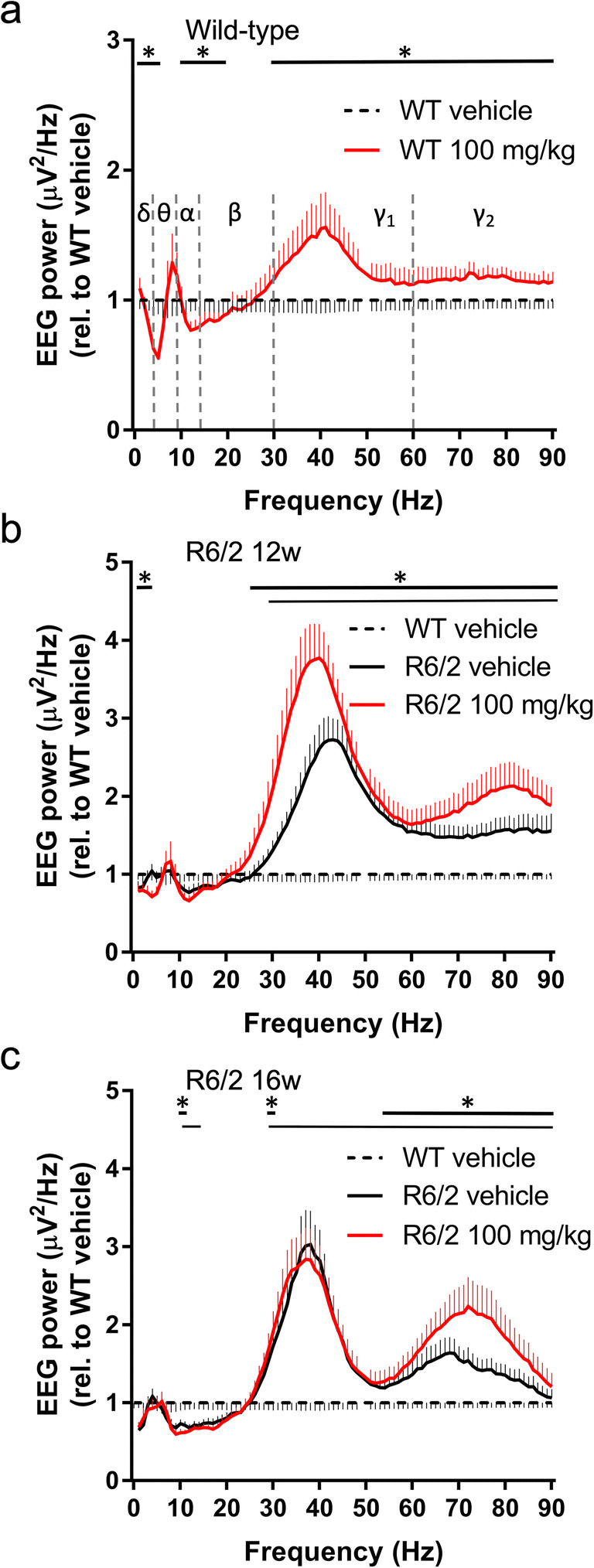
Effects of acute modafinil on the quantitative EEG during wakefulness in R6/2 and wild-type mice. EEG power from wild-type (WT, a) and R6/2 mice aged 12 weeks (b) and 16 weeks (c) was averaged in the first 2 h of the active phase after i.p. vehicle or 100 mg/kg modafinil treatment. Data (mean ± SEM) were normalized to the WT vehicle group (dashed line). Changes in the qEEG spectra following acute modafinil treatment was quantified across the spectrum in standard frequency ranges (δ [1–4Hz], θ [5–9Hz], α [10–14 Hz], β [15–30 Hz], γ1 [31–60 Hz], and γ2 [61–90 Hz]). Statistically significant differences (two-way ANOVA and Bonferroni post hoc comparisons, *p < 0.05) are shown by thick black bars (modafinil vs. vehicle treatments) and by thin black bars (vehicle treated WT vs. R6/2 mice) above the graphs

To visualize the genotype-specific and modafinil (100 mg/kg)-induced changes, all EEG power spectra were normalized to the WT vehicle group (Fig. 3). Modafinil had differential effect on the spectral power across multiple frequencies. Significant effects of modafinil on the qEEG spectra (ANOVA ‘treatment’ results for the whole frequency range and/or results of Bonferroni post hoc tests in case of significant treatment × frequency interaction) are shown on the graphs in Fig. 3. For full statistical analyses, see Table S2–3. We also analysed EEG power in standard frequency bands. Administration of modafinil decreased delta (1–4 Hz) power in WT and R6/2 mice at 12 but not 16 weeks (Fig. 3). Whilst theta (5–9 Hz) power was suppressed in WT but not R6/2 mice, the peak frequency of theta increased in both WT and all R6/2 mice (Table S4). Alpha (10–14 Hz) power was reduced in WT and R6/2 mice at 16 weeks, but not in R6/2 mice at 12 weeks. For beta (15–30 Hz) power, the effect varied across the frequency range. For WT mice, the power at frequencies < 20 Hz decreased significantly, whilst power at frequencies > 29 Hz increased significantly (Fig. 3a). In R6/2 mice at 12 weeks, only the beta power at frequencies of 25–30 Hz increased in response to modafinil, whilst in R6/2 mice at 16 weeks, only the power of 29–30 Hz increased (Fig. 3b, c). Gamma power was analysed separated into two bands (low [31–60 Hz] and high [> 60 Hz]). Gamma power increases progressively as part of the phenotype in R6/2 mice (see vehicle-treated mice in Fig. 3b, c). Modafinil increased both low and high gamma power in WT and R6/2 mice at 12 weeks (Fig. 3a, b). In R6/2 mice at 16 weeks, only high gamma was increased in response to modafinil (Fig. 3c). Modafinil had no effect on power spectrum in the passive phase (13–18 h following drug administration) in WT and R6/2 mice (data not shown).

### Acute Modafinil Induces Delta Rebound in NREMS in WT and R6/2 Mice

**Fig. 4:**
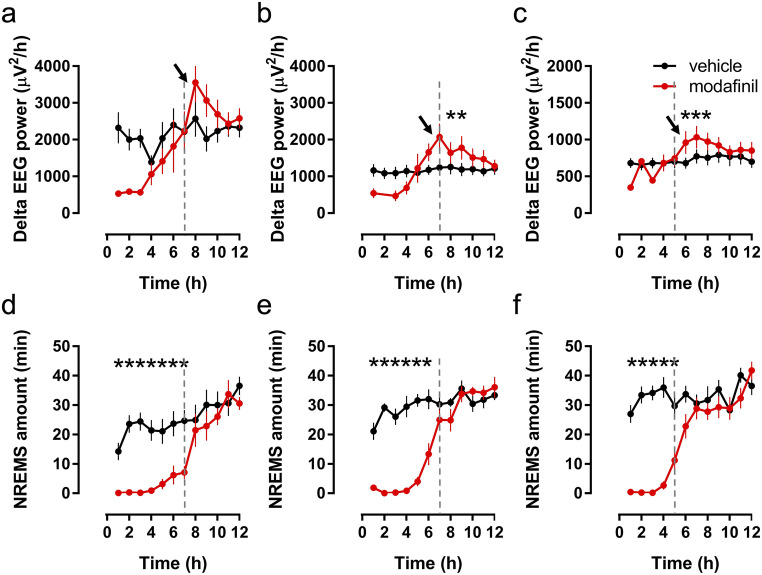
Delta power changes induced by modafinil during non-rapid eye movement sleep (NREMS) in R6/2 and wild-type mice. Delta power (1–4 Hz) was averaged for each hour after the injection of vehicle or modafinil (100 mg/kg, i.p.) at the beginning of active phase in wild-type (WT, a) or in R6/2 mice aged 12 weeks (b) and 16 weeks (c). Note that due to the robust NREMS-decreasing effect of modafinil (1–7 h) in WT and in R6/2 mice at 12 weeks of age (d and e, respectively), as well as (1–5 h) at 16 weeks of age (f), the average delta power data for these hours were not evaluated statistically. The vertical dashed lines show the end of the wake-promoting effect in each group. The arrows show the beginning of the ‘delta rebound’. Data are presented as mean ± SEM. *p < 0.05, compared to the relevant vehicle group

Once the wake-promoting effect of modafinil had dissipated in active phase (Fig. 4d–f), an immediate increase in the NREMS-delta power (delta rebound) was detected after treatment with 100 mg/kg dose of modafinil in the subsequent 3 h in WT and in R6/2 mice at 12 and 16 weeks (Fig. 4a–c; WT mice, *F*_(1.7)_ = 8.03, *p* = 0.0253; 12-week-old R6/2 mice, *F*_(1.7)_ = 11.59, *p* = 0.0086; 16-week-old R6/2 mice, *F*_(1.8)_ = 10.99, *p* = 0.0106). However, no rebound increase of NREMS was observed in any of the treatment groups. By contrast, in the passive phase (during the following 12 h), the 12-week-old R6/2 mice showed an increase in the amount of NREMS (*t*_(8)_ = 2.42, *p* = 0.0421), and also delta rebound during NREMS (*F*_(11.88)_ = 2.95, *p* = 0.0023) and this was only in response to the 100 mg/kg modafinil dose (see Table S1).

### Modafinil Maintains its Acute Wake-Promoting Effect Even After 9 Weeks of Chronic Treatment in R6/2 Mice

**Fig. 5:**
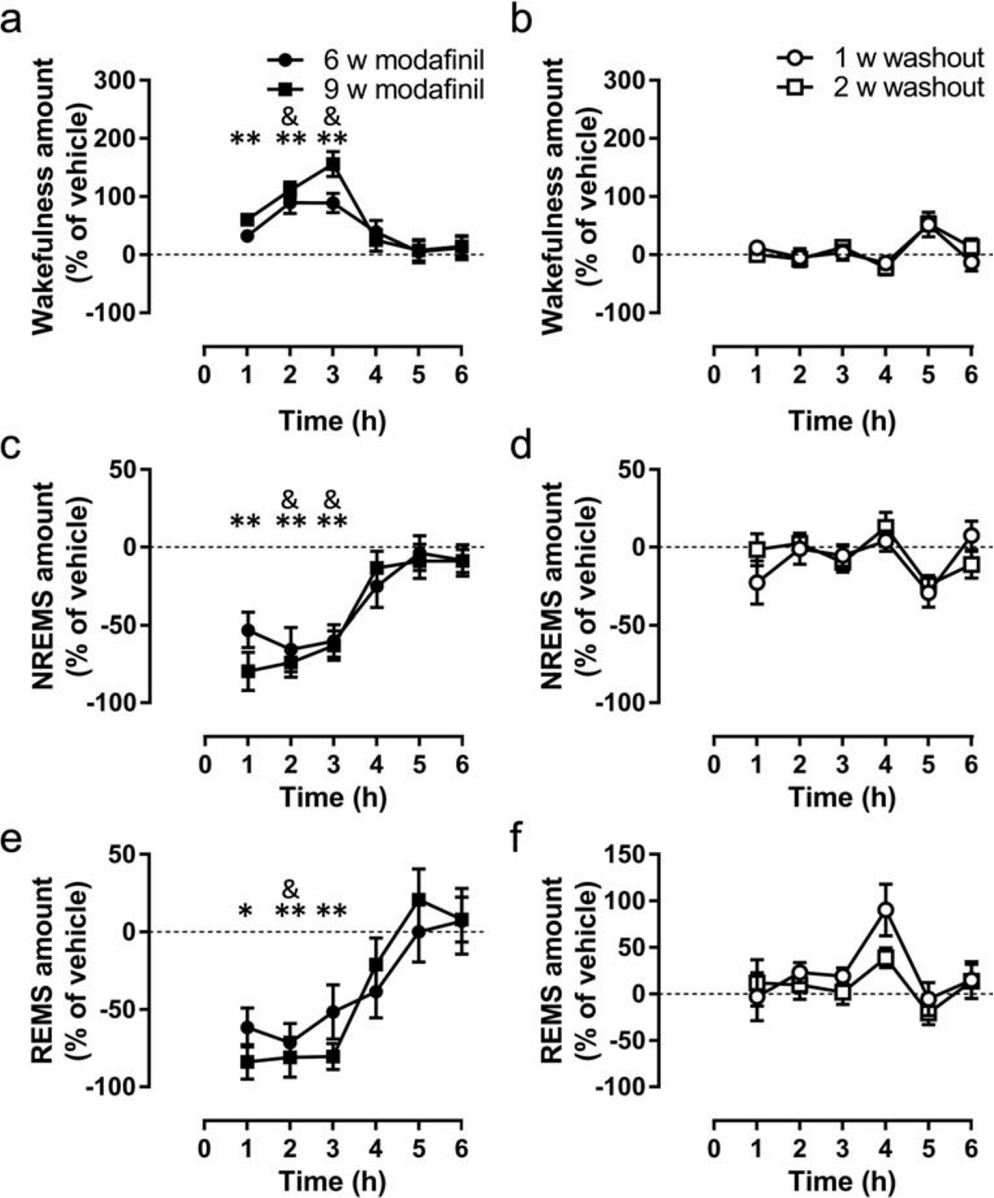
The effect of chronic treatment with modafinil on the pattern of sleep-wake cycle in R6/2 mice. Modafinil (64 mg/kg, per os) significantly changed the amount of wakefulness (a), non-rapid eye movement sleep (NREMS, b), and rapid eye movement sleep (REMS, c) after 6 and 9 weeks of treatment, although the effect reversed gradually following 1 or 2 weeks of washout (b, d, and f, respectively). The arrows indicate the time of the treatment at the beginning of active phase. Data are presented as mean ± SEM. ^&^*p* < 0.01 after 6 weeks of treatment, and *p < 0.05 and **p < 0.01 after 9 weeks of treatment, compared to the relevant vehicle groups

For the chronic regime, modafinil (64 mg/kg) was administered daily for 63 days at the beginning of the active phase starting at 6 weeks of age. In chronically treated R6/2 mice, the wake-promoting effect of modafinil in active phase lasted ~ 3 h after both 6 (Fig. 5a; drug effect, *F*_(1,16)_ = 25.57, *p* = 0.0001; interaction, *F*_(5,80_ = 2.89, *p* = 0.0188) and 9 weeks of treatment (Fig. 5a; drug: *F*_(1,16)_ = 81.32, *p* < 0.0001, interaction: *F*_(5,80_ = 5.70, *p* = 0.0002). The increase in wakefulness was mostly at the expense of NREMS both after 6 (Fig. 5b, drug: *F*_(1,16)_ = 26.03, *p* = 0.0001, interact.: *F*_(5,80_ = 2.96, *p* = 0.0169) and 9 weeks of treatment (drug: *F*_(1,16)_ = 71.86, *p* < 0.0001, interaction: *F*_(5,80_ = 4.97, *p* = 0.0005). Modafinil also suppressed REMS. This effect was more powerful after treatment for 9 weeks (Fig. 5c, drug: *F*_(1,16)_ = 26.29, *p* = 0.0001, interaction: *F*_(5,80)_ = 6.11, *p* < 0.0001) than it was after 6 weeks (Fig. 5c, drug: *F*_(1,16)_ = 13.30, *p* = 0.0022). Following washout, no effect of modafinil on the sleep-wake pattern was seen (Fig. 5b, d and f) apart from a small effect on REMS bout duration at 2 weeks after washout (Table S5).

### Chronic Modafinil Treatment Differentially Suppresses the EEG Power in Sleep and Wakefulness in R6/2 Mice

**Fig. 6:**
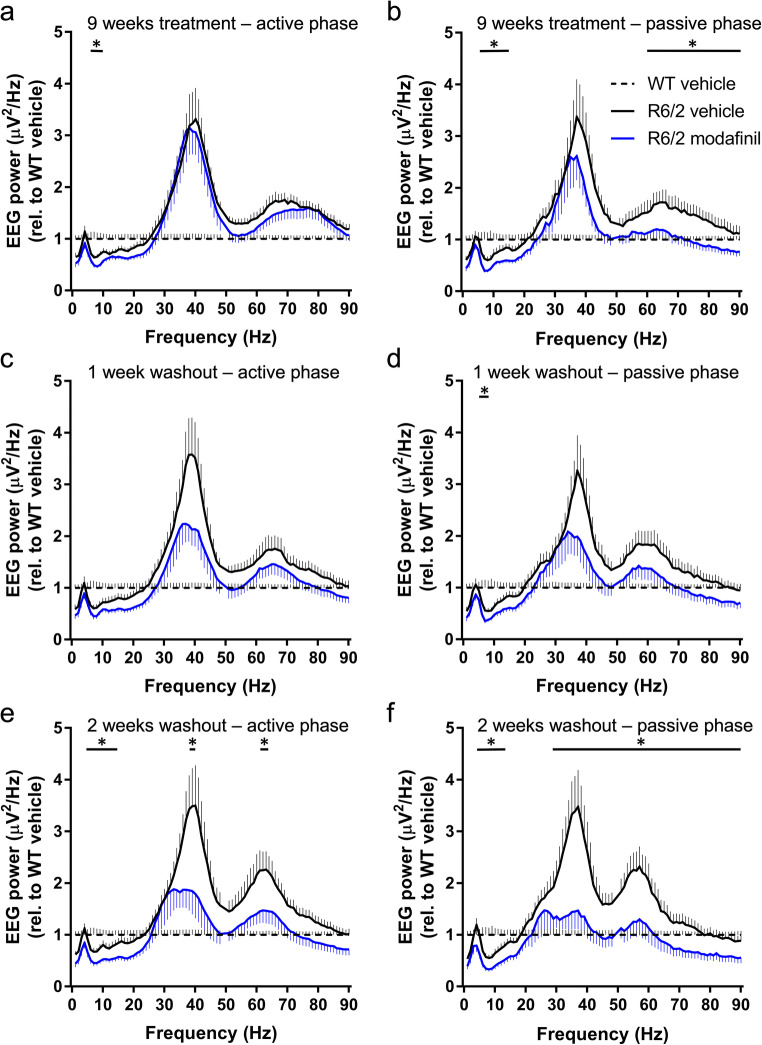
The effect of chronic modafinil treatment on the quantitative electroencephalography (EEG) in R6/2 mice measured during wakefulness. EEG spectra of vehicle- (solid black line) or modafinil (solid blue line)-treated groups during the first 2 h of the active (a, c, and e) and the passive phase (b, d, and f), after 9 weeks of treatment (a and b) and following 1 week (c and d) or 2 weeks of washout (e and f). Data are presented as mean ± SEM in 1 Hz bins, normalized to the WT vehicle group (dashed line). Changes in the qEEG spectra following chronic modafinil treatment were quantified across the spectrum in standard frequency ranges (δ [1–4Hz], θ [5–9Hz], α [10–14 Hz], β [15–30 Hz], γ1 [31–60 Hz], and γ2 [61–90 Hz]). Statistically significant differences (two-way ANOVA and Bonferroni post hoc comparisons, *p < 0.05) are shown by black bars (modafinil vs. vehicle treatments) above the graphs

After 9 weeks of treatment, there was a small but significant decrease in theta power in the active phase (Fig. 6a). Interestingly, in the passive phase, 12 h after drug administration (Fig. 6b), theta, alpha, and high gamma power were reduced in the treated mice. After 1 week of drug washout, the reduction in theta power in passive phase persisted (Fig. 6c, d). Following 2 weeks of washout, when R6/2 mice were at a late stage of disease, there were striking differences in EEG power between modafinil- and vehicle-treated mice in both active and passive phases. EEG power in modafinil-treated mice was significantly lower in theta, alpha, and both gamma frequency bands (Fig. 6e, f). That is, vehicle-treated mice showed abnormalities in EEG spectra that are suppressed in the modafinil-treated group, even 2 weeks after drug treatment stopped.

In NREM sleep, 9 weeks of modafinil treatment reduced the EEG power of delta, theta, alpha, and beta frequency bands (Fig. S2a). After 1 week of washout, this effect was visible but no longer significant (Fig. S2c). After 2 weeks of washout, the entire NREMS qEEG spectra was increased in the vehicle-treated R6/2 mice, consistent with progression of disease. Interestingly, it remained suppressed in the modafinil-treated mice (Fig. S2e).

In REMS, the power of alpha and both low and high gamma were suppressed after 9 weeks of treatment. This effect persisted for the 2 weeks of washout. After 2 weeks of washout, the difference between the EEG power of vehicle- and modafinil-treated R6/2 mice was extensive, affecting all frequency bands apart from delta (Fig. S2b, d and f). Full statistical analyses of the chronic modafinil-induced qEEG effects are in Table S6–11. Six weeks of treatment induced similar changes on the qEEG during wakefulness, NREMS, and REMS to that observed after 9 weeks of treatment (Fig. S3a-d, and Table S12–13).

## Discussion

Here we report for the first time that modafinil improves sleep-wake pattern and abnormalities of qEEG in R6/2 HD mice. Acute modafinil restores the amount of wakefulness and NREMS to the WT level, even in the late stage of disease. The wake-promoting effect of modafinil is reflected as a decrease in power of low (< 12 Hz) and an increase in power of high (> 24 Hz)-frequency EEG oscillations. Acute modafinil reduces the pathologically increased REMS in active phase and consolidates sleep in the passive phase in R6/2 mice. In chronically treated R6/2 mice, modafinil maintains its acute wake-promoting and REMS-reducing effect. Moreover, it causes long-lasting suppression of EEG power including the pathologically increased gamma seen in R6/2 mice.

The sleep-wake cycle is regulated by complex homeostatic and circadian processes [42], and involves several cortical and subcortical structures [43–45]. In HD patients, although the early neurodegeneration affects the medium-sized spiny neurons in the striatum, eventually neurodegeneration is widespread, affecting multiple parts of the brain including the circuits and neurotransmitter systems involved in the regulation of sleep and wake [46, 47]. Together, these changes probably contribute to the sleep disturbance in HD. In R6/2 mice, the sleep-wake abnormalities are characterised by a breakdown in circadian regulation with an increase in wake during the passive phase and sleep during the active phase [11, 12]. Here, acute modafinil promoted wakefulness at the expense of sleep in a similar manner in both WT and R6/2 mice, although in R6/2 mice at 16 weeks, the response to the highest dose (100 mg/kg) was shorter than that seen at 12 weeks. The wake-promoting effect of the daily doses of modafinil persisted in the chronically treated R6/2 mice, suggesting that there was no desensitization to the effect of modafinil. Together these data suggest that the circuits and neurotransmitter systems crucial for the generation and maintenance of wakefulness [45] remain intact in R6/2 mice, although they show some changes in sensitivity with the progression of the disease. Modafinil consolidated the fragmented NREMS seen in R6/2 mice in the passive phase. This effect is particularly relevant, since it has been hypothesised that being unable to sustain wakefulness in the active phase contributes to poor sleep in HD [3]. Thus, although modafinil is wake-promoting, it may also have beneficial knock-on effects on sleep. A key feature of pathological changes in HD mice is that the amount of REMS increases progressively [11, 12]. Interestingly, acute modafinil had much less effect on REMS than it did on NREMS, with only the 100 mg/kg dose restoring REMS to the WT level, and only at 12 weeks. We speculate that the networks involved in the regulation of REMS are less sensitive to the effect of modafinil compared to those circuits involved in the regulation of wakefulness/NREMS.

Changes in the qEEG spectra following acute modafinil reflect its wake-promoting action. The effects of modafinil on the qEEG spectra were diverse and depended upon both the age of the mice and the dose regime. As shown previously in WT mice [39], acute modafinil reduced delta power in 12-week-old R6/2 mice, but not at 16 weeks of age, suggesting that cortical and thalamic circuits important for the generation of delta oscillations [48] are still intact in the young symptomatic age of R6/2 mice. In HD patients, the subjective feeling of sleepiness, reflected by increased delta power in wakefulness, is a progressive feature of the disease [6, 49, 50]. Thus, we can speculate that daytime sleepiness in HD may be improved via decreasing delta power by modafinil. The idea that these circuits are still intact in the R6/2 mice is supported by the emergence of delta rebound during NREMS. Delta power in NREMS is an indicator of homeostatic sleep drive that increases with accumulating sleep pressure [42]. Delta rebound in R6/2 mice is blunted following sleep deprivation [12], due to the impaired ability of the cortex to generate and propagate delta oscillations, possibly as a result of hyperexcitability of cortical pyramidal neurons that has been reported in R6/2 mouse brain slices [51]. Thus, its emergence after modafinil-induced wakefulness is particularly interesting since it supports the idea that, at least at 12 weeks, cortical circuits regulating delta generation are intact, but not operating normally in the untreated R6/2 mice.

Acute modafinil increased beta, low, and high gamma power in WT and also in 12-week-old R6/2 mice in line with studies reporting EEG-desynchronising effects of modafinil [52, 53]. In HD, increased beta and gamma power are phenotypic markers in both human [3] and (R6/2 and Q175) mouse models of HD [11, 12, 16]. The effect of acute modafinil on R6/2 mice was seen over and above the pathologically increased power seen in those spectral ranges. However, by 16 weeks, only the power of high gamma and a narrow band of beta was increased significantly, suggesting that by this age the neuronal circuits generating beta and low gamma frequencies in R6/2 mice had lost their sensitivity to modafinil. Although 9 weeks of chronic modafinil treatment produced no striking changes on the qEEG in active phase, passive phase data revealed significant changes. For example, whilst high gamma power of vehicle-treated R6/2 mice increased pathologically, in the chronic modafinil-treated mice the abnormally high gamma was suppressed. Moreover, after 2 weeks of washout, suppression of both the low and high gamma power persisted in the previously treated mice. Since in human HD increased gamma power has been suggested to be a component of the uncontrollable choreatic movements [54], reduction of this might be beneficial in HD.

The effect of chronic modafinil on beta oscillations was particularly interesting, as despite chronic modafinil having no effect on beta power, after 2 weeks washout, the abnormal changes in beta did not emerge in the chronic modafinil group. This suggests that chronic modafinil might have supressed the pathological remodelling of circuits responsible for the generation of abnormal beta oscillations. The differential response of beta, low, and high gamma to the effect of acute modafinil in 12- and 16-week-old R6/2 mice also supports the idea that progressive neurodegenerative changes modify the sensitivity of these networks differentially. Also, these results suggest that circuits giving rise to beta and gamma oscillations are modulated independently. Indeed, in humans, suppression of beta power has been suggested to play an antikinetic role in movement regulation [55, 56], whilst the amplitude of low and high gamma oscillations is modulated related to the phase of the gait cycle [56].

In both HD patients [49] and R6/2 mice [12], there is a pathological decrease in theta power as well as a decrease in the peak frequency of theta oscillations in the wake EEG. As seen in WT mice [39], acute modafinil increased the peak theta frequency in R6/2 mice at both 12 and 16 weeks, suggesting that, as with the circuits generating gamma, those generating theta remain responsive to the acute effect of modafinil. Theta is a fundamental brain rhythm mediating the information flow through the hippocampus, thalamus, and frontal cortex [57] that has been linked to cognitive functions in both rodents [58] and humans [59]. Thus, restoring or further increasing the frequency of theta may mediate a beneficial effect of modafinil on cognition in HD.

In the EEG signature of HD, changes in alpha power may reflect dysfunction of thalamic networks [49, 60]. The change in alpha power is usually a decrease, however, increased alpha has also been reported as an electrophysiological correlate of sustained motor activity during NREMS in HD patients [61]. By 16 weeks, the pathological decrease in alpha power emerged in R6/2 mice. Interestingly, acute modafinil decreased it further at this age. The suppressive effect of modafinil on alpha was also significant in the chronic treatment group and was maintained for 2 weeks of washout. As alpha is linked to a number of different brain functions, including sensory and memory processes (reviewed in [62]), this needs further studies to clarify how sustained suppression of alpha might affect these behavioural correlates.

Another property of modafinil that may contribute to a beneficial effect on sleep is the amelioration of neuroinflammation in sleep-deprived rats by reducing the level of pro-inflammatory cytokines [63]. This is particularly relevant, as the accumulation of pro-inflammatory cytokines has been demonstrated to induce neuroinflammation in HD [64]. Moreover, modafinil has been shown to promote hippocampal neurogenesis [65] and synaptic plasticity [66, 67], and its possible neuroprotective role has also been reported against methamphetamine induced striatal toxicity [68]. Thus, there are multiple mechanisms whereby modafinil might be beneficial in HD.

It is clear that modafinil has a complex action on different components of the qEEG and that not all of the actions of modafinil can be explained by our current understanding of the circuitry underlying HD. It is possible that some of the qEEG changes in the R6/6 mice are compensatory and therefore it may not be desirable to reverse them. What has emerged from our study is that different parts of the abnormal EEG in R6/2 mice are modulated differentially by modafinil. Furthermore, some of these effects are sustained long after the drug would be predicted to have disappeared from the system. It is possible that chronic modafinil treatment prevented pathological changes in the R6/2 mouse brain. Alternatively, modafinil may have modulated other changes occurring in the R6/2 brain such that even after cessation of the treatment, lasting benefits were seen. That is, modafinil may be masking the ongoing pathology. Regardless of the underlying mechanism, it is interesting that the beneficial effects of modafinil persisted 2 weeks after the treatment. The complexities of modafinil action are not entirely unexpected. All these transmitter systems are involved in regulation of sleep and wake [45] as well as in HD pathology (for references, see [1]). Indeed, it is not known precisely which pathways generate HD symptoms such as motor, cognitive, and sleep disorder. The complexity of the pathways modulating behaviour and their interactions are likely to be increased in the dynamically degenerating brain. Studying the effects of a drug such as modafinil on EEG may give some insight into this interrelationship.

## Limitation of the Study

Our data show that chronic modafinil treatment evoked long-lasting changes on the EEG powers in R6/2 mice, suggesting that modafinil had beneficial effects. However, given that we were not able to collect parallel data from WT mice, we cannot determine if these changes are specific to the R6/2 mice, nor can we say anything about the possible long-term effect on EEG spectra of WT mice.

## Conclusions

Modafinil has acute beneficial effects on the sleep-wake pattern of R6/2 mice that suggests that it may be useful clinically for treating sleep-wake disorder in HD. Chronic modafinil induced both short- and long-lasting changes in qEEG and prevents the manifestation of pathological changes in the EEG spectrum. Whilst modafinil acted clearly as a wake-promoting agent in R6/2 mice, its complex action on qEEG shows that modafinil differentially affects brain systems likely to be involved not only in the regulation of sleep and wakefulness but also in other higher brain functions. A better understanding of these actions is needed to use modafinil to best effect in patients with complex neurological disorders.

## Electronic Supplementary Material

Supplemental Figure 1 (PNG 5360 kb)

High Resolution (TIFF 551 kb)

Supplemental Figure 2 (PNG 5360 kb)

High Resolution (TIFF 1082 kb)

Supplemental Figure 3 (PNG 5360 kb)

High Resolution (TIFF 824 kb)

ESM 1 (DOCX 1.01 MB)

ESM 2 (DOCX 47.6 KB)

ESM 3 (PDF 1224 kb)

## References

[1] Bates and Tabrizi and J. Huntington’s Disease, 4th ed. 10.1093/med/9780199929146.001.0001

[2] Goodman AOG, Morton AJ, Barker RA (2010). Identifying sleep disturbances in Huntington’s disease using a simple disease-focused questionnaire. PLoS Curr.

[3] Lazar AS, Panin F, Goodman AOG (2015). Sleep deficits but no metabolic deficits in premanifest Huntington’s disease. Ann Neurol.

[4] Piano C, Losurdo A, Della Marca G (2015). Polysomnographic Findings and Clinical Correlates in Huntington Disease: A Cross-Sectional Cohort Study. Sleep.

[5] Arnulf I, Nielsen J, Lohmann E (2008). Rapid eye movement sleep disturbances in Huntington disease. Arch Neurol.

[6] Videnovic A, Leurgans S, Fan W, Jaglin J, Shannon KM (2009). Daytime somnolence and nocturnal sleep disturbances in Huntington disease. Park Relat Disord.

[7] Piano C, Mazzucchi E, Bentivoglio AR (2017). Wake and Sleep EEG in Patients with Huntington Disease. Clin EEG Neurosci.

[8] Aziz NA, Anguelova GV, Marinus J, Lammers GJ, Roos RAC (2010). Sleep and circadian rhythm alterations correlate with depression and cognitive impairment in Huntington’s disease. Park Relat Disord.

[9] Morton AJ (2013). Circadian and sleep disorder in Huntington’s disease. Exp Neurol.

[10] Nguyen L, Bradshaw JL, Stout JC, Croft RJ, Georgiou-Karistianis N (2010). Electrophysiological measures as potential biomarkers in Huntington’s disease: Review and future directions. Brain Res Rev.

[11] Kantor S, Szabo L, Varga J, Cuesta M, Morton AJ (2013). Progressive sleep and electroencephalogram changes in mice carrying the Huntington’s disease mutation. Brain.

[12] Fisher SP, Black SW, Schwartz MD (2013). Longitudinal analysis of the electroencephalogram and sleep phenotype in the R6/2 mouse model of Huntington’s disease. Brain.

[13] Pignatelli M, Lebreton F, Cho YH, Leinekugel X (2012). “Ectopic” theta oscillations and interictal activity during slow-wave state in the R6/1 mouse model of Huntington’s disease. Neurobiol Dis.

[14] Lebreton F, Cayzac S, Pietropaolo S, Jeantet Y, Cho YH (2015). Sleep physiology alterations precede plethoric phenotypic changes in R6/1 Huntington’s disease mice. PLoS One.

[15] Loh DH, Kudo T, Truong D, Wu Y, Colwell CS (2013). The Q175 Mouse Model of Huntington’s Disease Shows Gene Dosage- and Age-Related Decline in Circadian Rhythms of Activity and Sleep. PLoS One.

[16] Rothe T, Deliano M, Wójtowicz AM (2015). Pathological gamma oscillations, impaired dopamine release, synapse loss and reduced dynamic range of unitary glutamatergic synaptic transmission in the striatum of hypokinetic Q175 Huntington mice. Neuroscience.

[17] Fisher SP, Schwartz MD, Wurts-Black S (2016). Quantitative Electroencephalographic Analysis Provides an Early-Stage Indicator of Disease Onset and Progression in the zQ175 Knock-In Mouse Model of Huntington’s Disease. Sleep.

[18] Mangiarini L, Sathasivam K, Seller M (1996). Exon I of the HD gene with an expanded CAG repeat is sufficient to cause a progressive neurological phenotype in transgenic mice. Cell.

[19] Lione LA, Carter RJ, Hunt MJ (1999). Selective discrimination learning impairments in mice expressing the human Huntington’s disease mutation. J Neurosci.

[20] Carter RJ, Lione LA, Humby T (1999). Characterization of progressive motor deficits in mice transgenic for the human Huntington’s disease mutation. J Neurosci.

[21] Pallier PN, Morton AJ (2009). Management of sleep/wake cycles improves cognitive function in a transgenic mouse model of Huntington’s disease. Brain Res.

[22] Wulff K, Gatti S, Wettstein J, Foster R (2010). Sleep and circadian rhythm disruption in psychiatric and neurodegenerative disease. Nat Rev Neurosci.

[23] Baker CR, Domínguez DJF, Stout JC (2016). Subjective sleep problems in Huntington’s disease: A pilot investigation of the relationship to brain structure, neurocognitive, and neuropsychiatric function. J Neurol Sci.

[24] Blackwell AD, Paterson NS, Barker RA, Robbins TW, Sahakian BJ (2008). The effects of modafinil on mood and cognition in Huntington’s disease. Psychopharmacology (Berl).

[25] Battleday RM, Brem AK (2015). Modafinil for cognitive neuroenhancement in healthy non-sleep-deprived subjects: A systematic review. Eur Neuropsychopharmacol.

[26] Herzog-Krzywoszanska R, Krzywoszanski L (2019). Sleep Disorders in Huntington’s Disease. Front Psychiatry.

[27] Thorpy MJ (2015). Update on Therapy for Narcolepsy. Curr Treat Options Neurol.

[28] Rodrigues TM, Caldas AC, Ferreira JJ (2016). Pharmacological interventions for daytime sleepiness and sleep disorders in Parkinson’s disease: Systematic review and meta-analysis. Park Relat Disord.

[29] Minzenberg MJ, Carter CS (2008). Modafinil: A review of neurochemical actions and effects on cognition. Neuropsychopharmacology.

[30] Murillo-Rodríguez E, Barciela Veras A, Barbosa Rocha N, Budde H, Machado S (2018). An Overview of the Clinical Uses, Pharmacology, and Safety of Modafinil. ACS Chem Neurosci.

[31] Randall DC, Shneerson JM, File SE (2005). Cognitive effects of modafinil in student volunteers may depend on IQ. Pharmacol Biochem Behav.

[32] Müller U, Rowe JB, Rittman T (2013). Effects of modafinil on non-verbal cognition, task enjoyment and creative thinking in healthy volunteers. Neuropharmacology.

[33] Minzenberg MJ, Yoon JH, Cheng Y, Carter CS (2016). Sustained Modafinil Treatment Effects on Control-Related Gamma Oscillatory Power in Schizophrenia. Neuropsychopharmacology.

[34] Lees J, Michalopoulou PG, Lewis SW (2017). Modafinil and cognitive enhancement in schizophrenia and healthy volunteers: The effects of test battery in a randomised controlled trial. Psychol Med.

[35] Turner D (2006). A review of the use of modafinil for attention-deficit hyperactivity disorder. Expert Rev Neurother.

[36] Abolfazli R, Hosseini M, Ghanizadeh A (2011). Double-blind randomized parallel-group clinical trial of efficacy of the combination fluoxetine plus modafinil versus fluoxetine plus placebo in the treatment of major depression. Depress Anxiety.

[37] Rasetti R, Mattay VS, Stankevich B (2010). Modulatory effects of modafinil on neural circuits regulating emotion and cognition. Neuropsychopharmacology.

[38] Paulsen JS, Nehl C, Hoth KF (2005). Depression and stages of Huntington’s disease. J Neuropsychiatry Clin Neurosci.

[39] Willie JT, Renthal W, Chemelli RM (2005). Modafinil more effectively induces wakefulness in orexin-null mice than in wild-type littermates. Neuroscience.

[40] Wisor JP, Eriksson KS (2005). Dopaminergic-adrenergic interactions in the wake promoting mechanism of modafinil. Neuroscience.

[41] Wood NI, Glynn D, Morton AJ (2011). “Brain training” improves cognitive performance and survival in a transgenic mouse model of Huntington’s disease. Neurobiol Dis.

[42] Borbely AA (1982). A two process model of sleep regulation. Hum Neurobiol.

[43] Saper CB, Chou TC, Scammell TE (2001). The sleep switch: Hypothalamic control of sleep and wakefulness. Trends Neurosci.

[44] Saper CB, Scammell TE, Lu J (2005). Hypothalamic regulation of sleep and circadian rhythms. Nature.

[45] Scammell TE, Arrigoni E, Lipton JO (2017). Neural Circuitry of Wakefulness and Sleep. Neuron.

[46] Vonsattel JP, Myers RH, Stevens TJ (1985). Neuropathological classification of huntington’s disease. J Neuropathol Exp Neurol.

[47] Rosas HD, Salat DH, Lee SY (2008). Complexity and heterogeneity: What drives the ever-changing brain in Huntington’s disease?. Ann N Y Acad Sci.

[48] Amzica F, Steriade M (1998). Electrophysiological correlates of sleep delta waves. Electroencephalogr Clin Neurophysiol.

[49] Bylsma FW, Peyser CE, Folstein SE, Ross C, Brandt J (1994). EEG power spectra in huntington’s disease: Clinical and neuropsychological correlates. Neuropsychologia.

[50] Painold A, Anderer P, Holl AK (2010). Comparative EEG mapping studies in Huntington’s disease patients and controls. J Neural Transm.

[51] Cummings DM, Andre VM, Uzgil BO (2009). Alterations in Cortical Excitation and Inhibition in Genetic Mouse Models of Huntington’s Disease. J Neurosci.

[52] Hasan S, Pradervand S, Ahnaou A (2009). How to keep the brain awake the complex molecular pharmacogenetics of wake promotion. Neuropsychopharmacology.

[53] Chen CR, Yang SR, Liu YY (2013). Roles of Adrenergic α1 and Dopamine D1 and D2 Receptors in the Mediation of the Desynchronization Effects of Modafinil in a Mouse EEG Synchronization Model. PLoS One.

[54] Ferrea S, Groiss SJ, Elben S (2018). Pallidal deep brain stimulation in juvenile Huntington’s disease: local field potential oscillations and clinical data. J Neurol.

[55] Jenkinson N, Brown P (2011). New insights into the relationship between dopamine, beta oscillations and motor function. Trends Neurosci.

[56] Seeber M, Scherer R, Wagner J, Solis-Escalante T, Müller-Putz GR (2014). EEG beta suppression and low gamma modulation are different elements of human upright walking. Front Hum Neurosci.

[57] Kirk IJ, Mackay JC (2003). The role of theta-range oscillations in synchronising and integrating activity in distributed mnemonic networks. Cortex.

[58] Vinogradova OS (1995). Expression, control, and probable functional significance of the neuronal theta-rhythm. Prog Neurobiol.

[59] Raghavachari S, Kahana MJ, Rizzuto DS (2001). Gating of Human Theta Oscillations by a Working Memory Task. J Neurosci.

[60] Bellotti R, De Carlo F, Massafra R, de Tommaso M, Sciruicchio V (2004). Topographic classification of EEG patterns in Huntington’s disease. Neurol Clin Neurophysiol.

[61] Piano C, Mazzucchi E, Bentivoglio AR (2016). Wake and Sleep EEG in Patients With Huntington Disease An eLORETA Study and Review of the Literature. Clin EEG Neurosci.

[62] Klimesch W, Sauseng P, Hanslmayr S (2007). EEG alpha oscillations: The inhibition-timing hypothesis. Brain Res Rev.

[63] Wadhwa M, Chauhan G, Roy K (2018). Caffeine and Modafinil Ameliorate the Neuroinflammation and Anxious Behavior in Rats during Sleep Deprivation by Inhibiting the Microglia Activation. Front Cell Neurosci.

[64] Staal RGW, Möller T. Neuroinflammation in Huntington’s disease. In: Neuroinflammation Neurodegener. Springer New York, pp 179–197

[65] Brandt MD, Ellwardt E, Storch A (2014). Short- and long-term treatment with modafinil differentially affects adult hippocampal neurogenesis. Neuroscience.

[66] Rao Y, Liu ZW, Borok E (2007). Prolonged wakefulness induces experience-dependent synaptic plasticity in mouse hypocretin/orexin neurons. J Clin Invest.

[67] Tsanov M, Lyons DG, Barlow S, González Reyes RE, O’Mara SM (2010). The psychostimulant modafinil facilitates water maze performance and augments synaptic potentiation in dentate gyrus. Neuropharmacology.

[68] Raineri M, González B, Rivero-Echeto C (2015). Differential Effects of Environment-Induced Changes in Body Temperature on Modafinil’s Actions Against Methamphetamine-Induced Striatal Toxicity in Mice. Neurotox Res.

