# Supplementary figures and images for: Wake-Promoting and EEG Spectral Effects of Modafinil After Acute or Chronic Administration in the R6/2 Mouse Model of Huntington’s Disease
Source: Neurotherapeutics. 2020 Apr 15;17(3):1075–86. doi: 10.1007/s13311-020-00849-y (PMC7609772; doi:10.1007/s13311-020-00849-y)

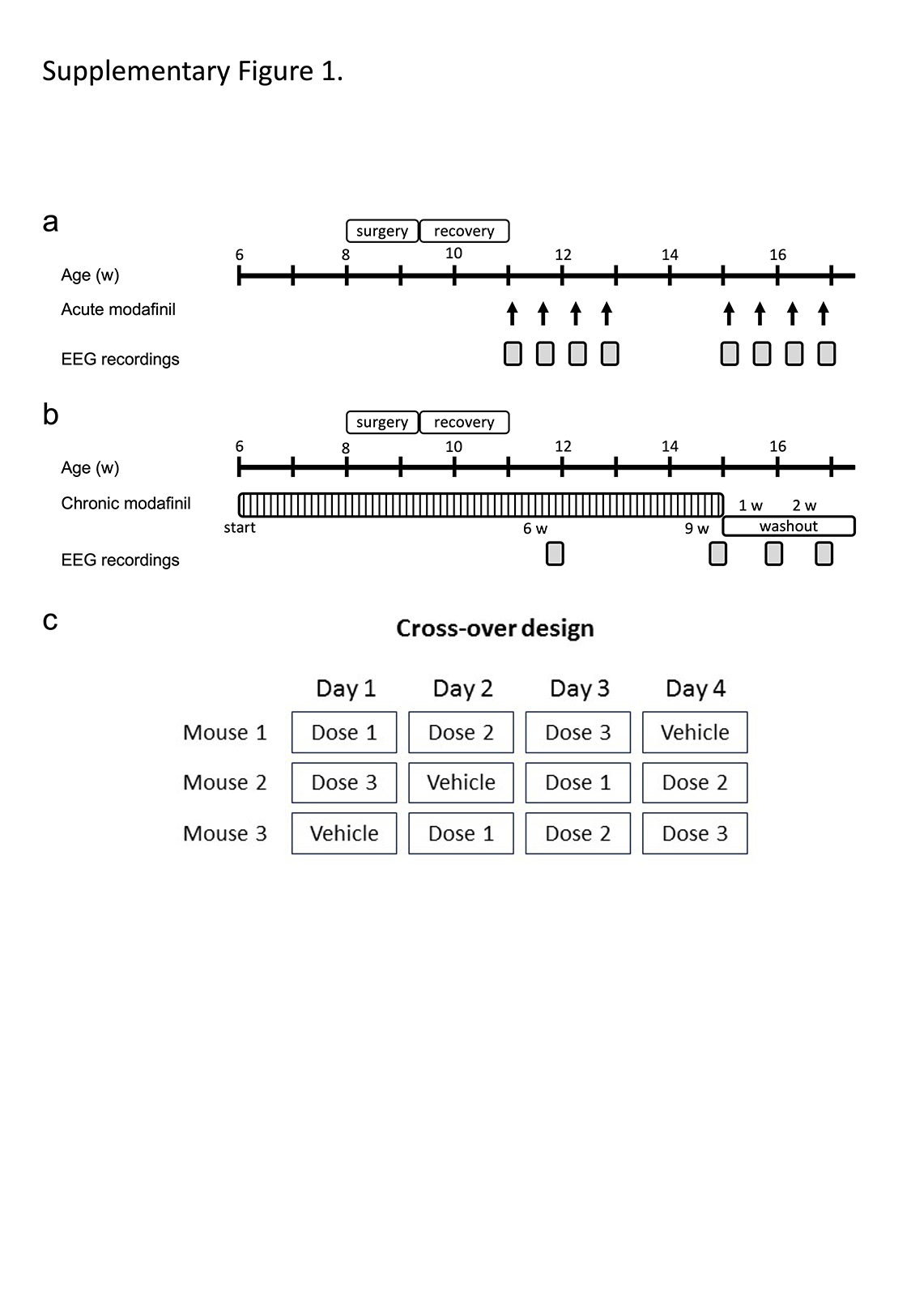

Supplement: Supplementary file 1 — (PNG 5360 kb) [file 13311_2020_849_Fig7_ESM.png]

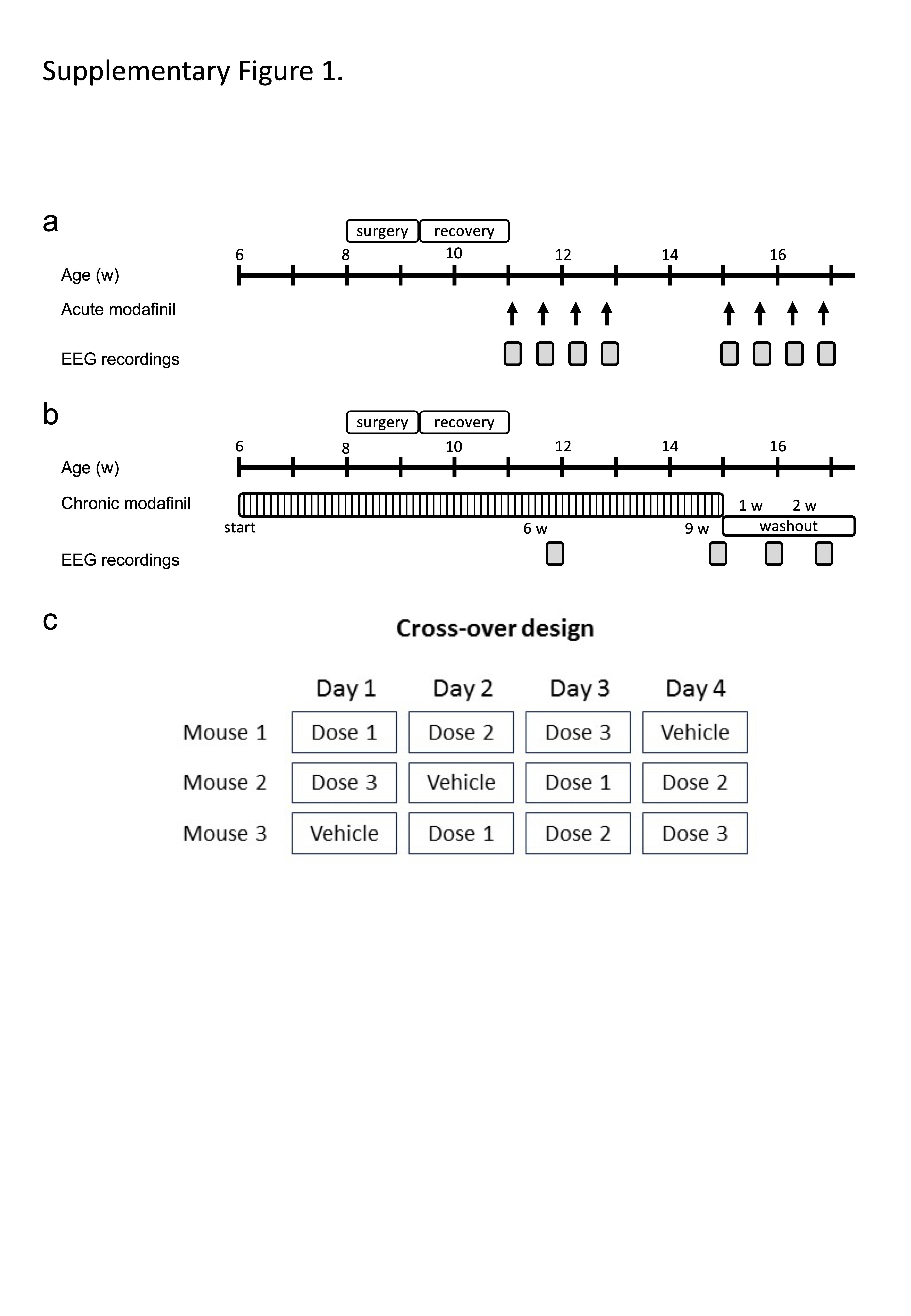

Supplement: Supplementary file 2 — High Resolution (TIFF 551 kb) [file 13311_2020_849_MOESM1_ESM.tiff]

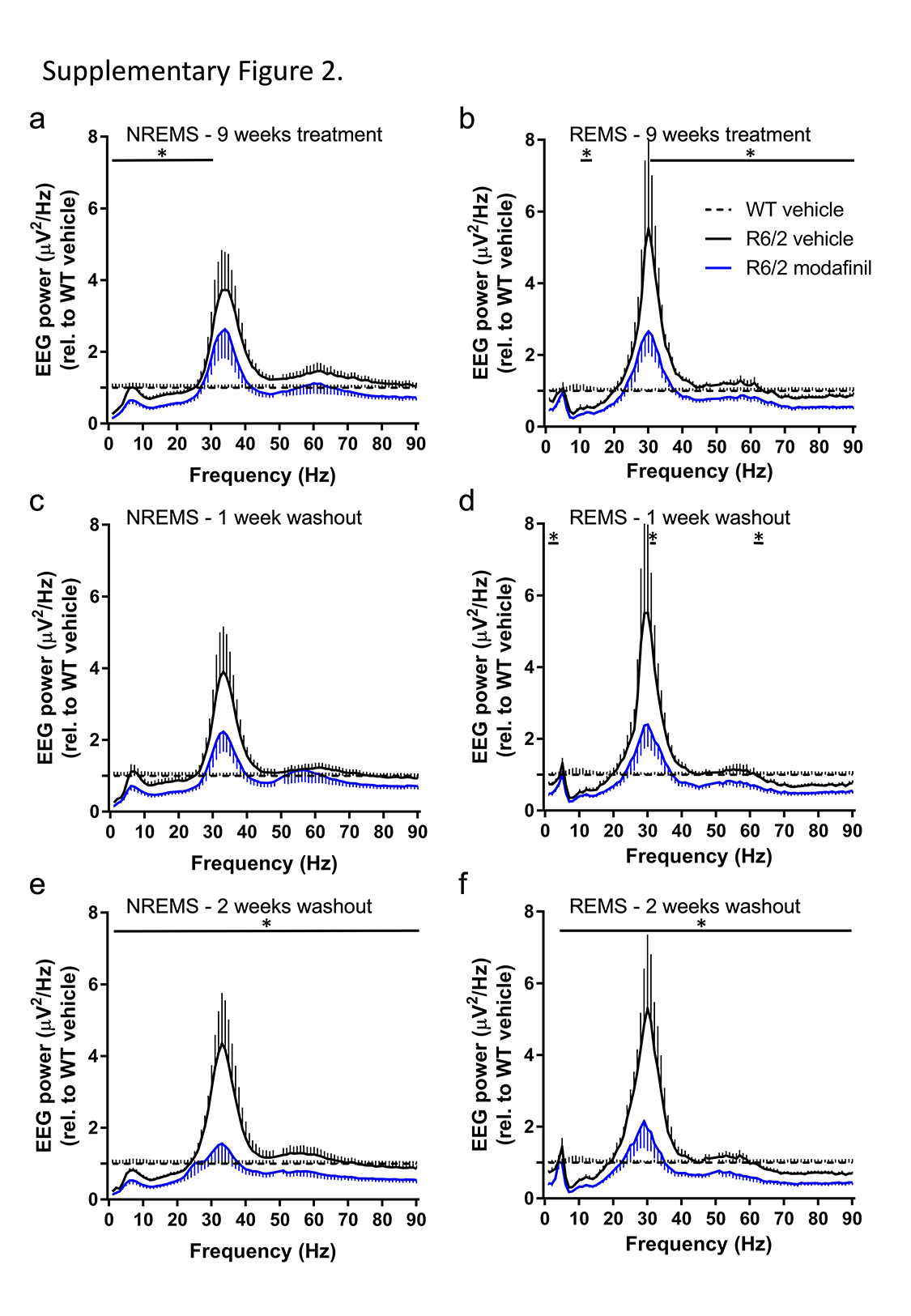

Supplement: Supplementary file 3 — (PNG 5360 kb) [file 13311_2020_849_Fig8_ESM.png]

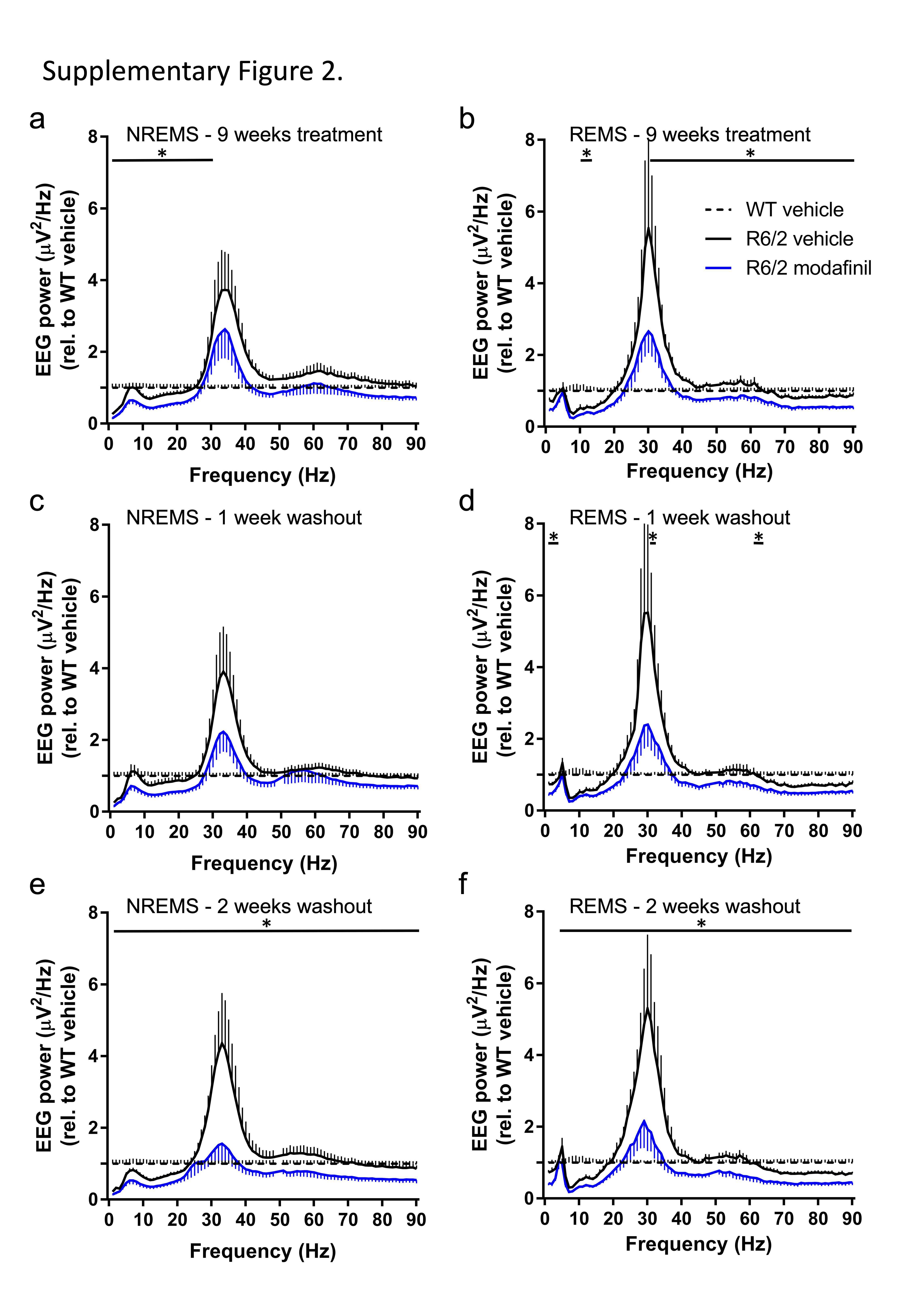

Supplement: Supplementary file 4 — High Resolution (TIFF 1082 kb) [file 13311_2020_849_MOESM2_ESM.tiff]

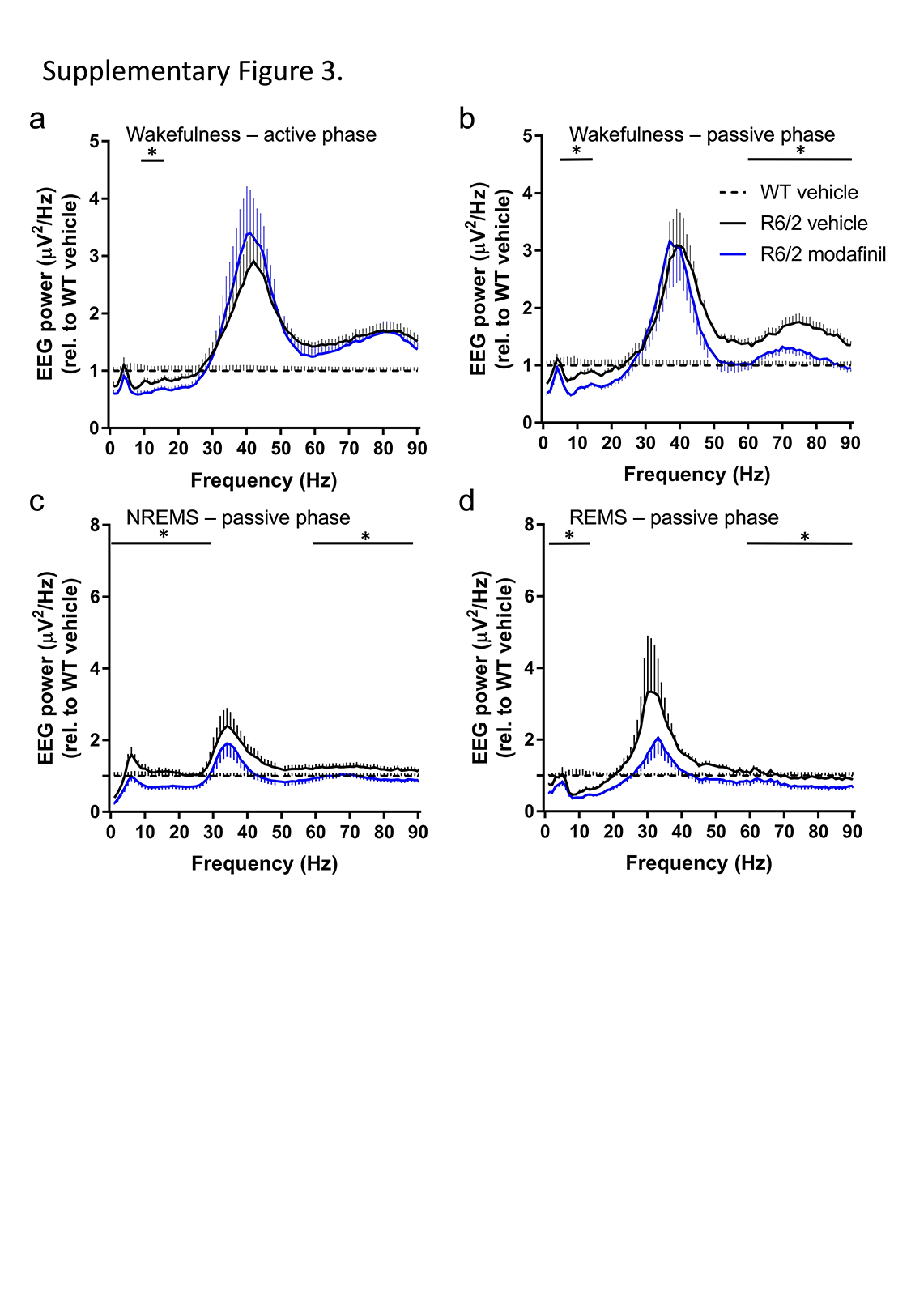

Supplement: Supplementary file 5 — (PNG 5360 kb) [file 13311_2020_849_Fig9_ESM.png]

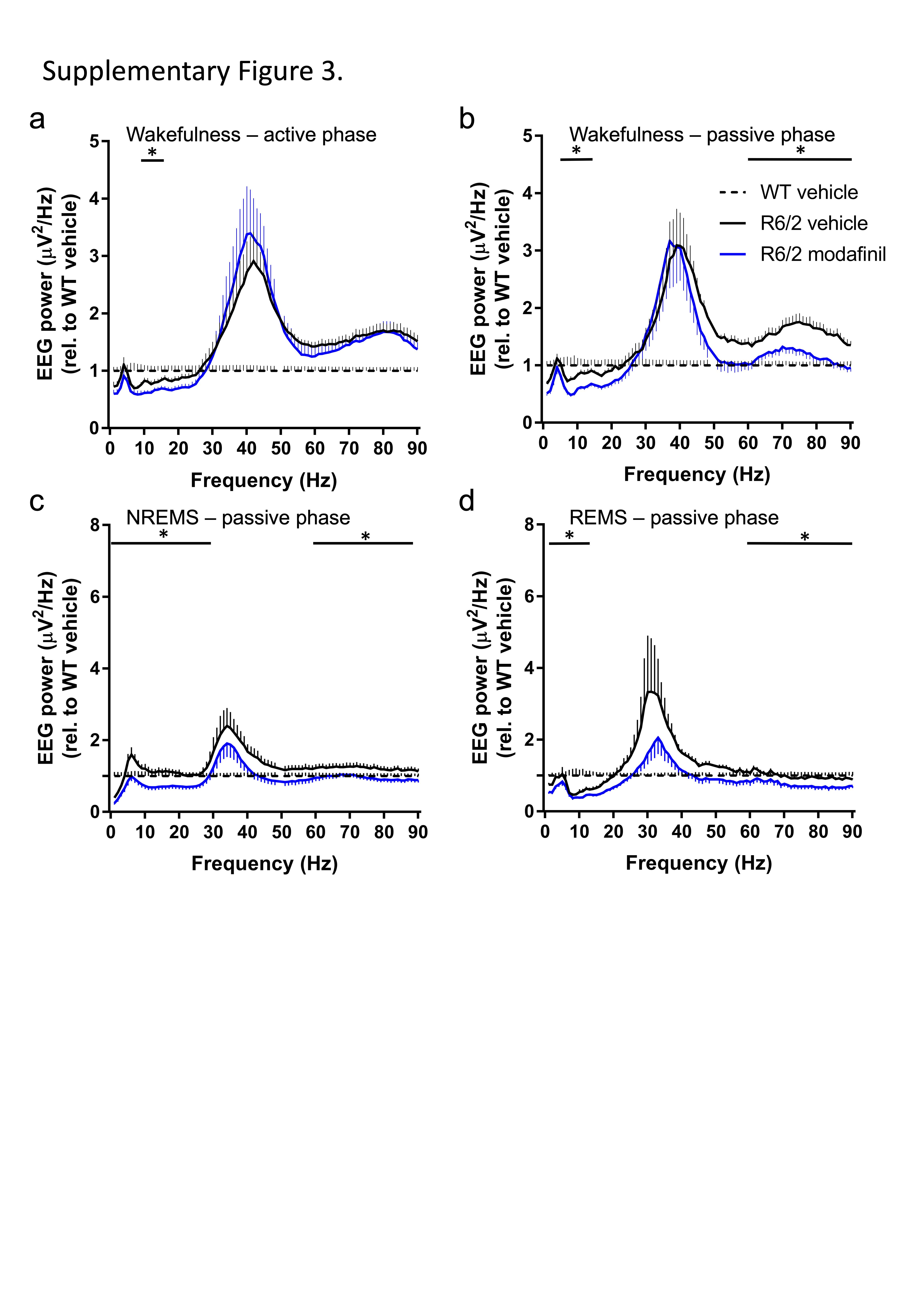

Supplement: Supplementary file 6 — High Resolution (TIFF 824 kb) [file 13311_2020_849_MOESM3_ESM.tiff]
