# Supplementary material for: Wake-Promoting and EEG Spectral Effects of Modafinil After Acute or Chronic Administration in the R6/2 Mouse Model of Huntington’s Disease
Source: Neurotherapeutics. 2020 Apr 15;17(3):1075–86. doi: 10.1007/s13311-020-00849-y (PMC7609772; doi:10.1007/s13311-020-00849-y)
Supplement: Supplementary file 7 — (DOCX 1.01 MB) [file 13311_2020_849_MOESM4_ESM.docx]

**Supplementary information**

**Wake-promoting and EEG spectral effects of modafinil after acute or chronic administration in the R6/2 mouse model of Huntington’s disease**

Szilvia Vas PhD^*^, Jackie M Casey^*^, Will T Schneider PhD^*^, Lajos Kalmar PhD†

and A Jennifer Morton, PhD, ScD^*‡^

^*^Department of Physiology, Development and Neuroscience, University of Cambridge, Downing Street, Cambridge, CB2 3DY, UK, †Department of Veterinary Medicine, University of Cambridge, Madingley Road, Cambridge, CB3 0ES, UK

^‡^Author for correspondence

A. Jennifer Morton,

Department of Physiology, Development and Neuroscience,

University of Cambridge,

Downing Street,

Cambridge CB2 3DY,

United Kingdom

Phone: +44 1223 334057

Fax: +44 1223 333840

E-mail: ajm41@cam.ac.uk

# Supplementary figures

**
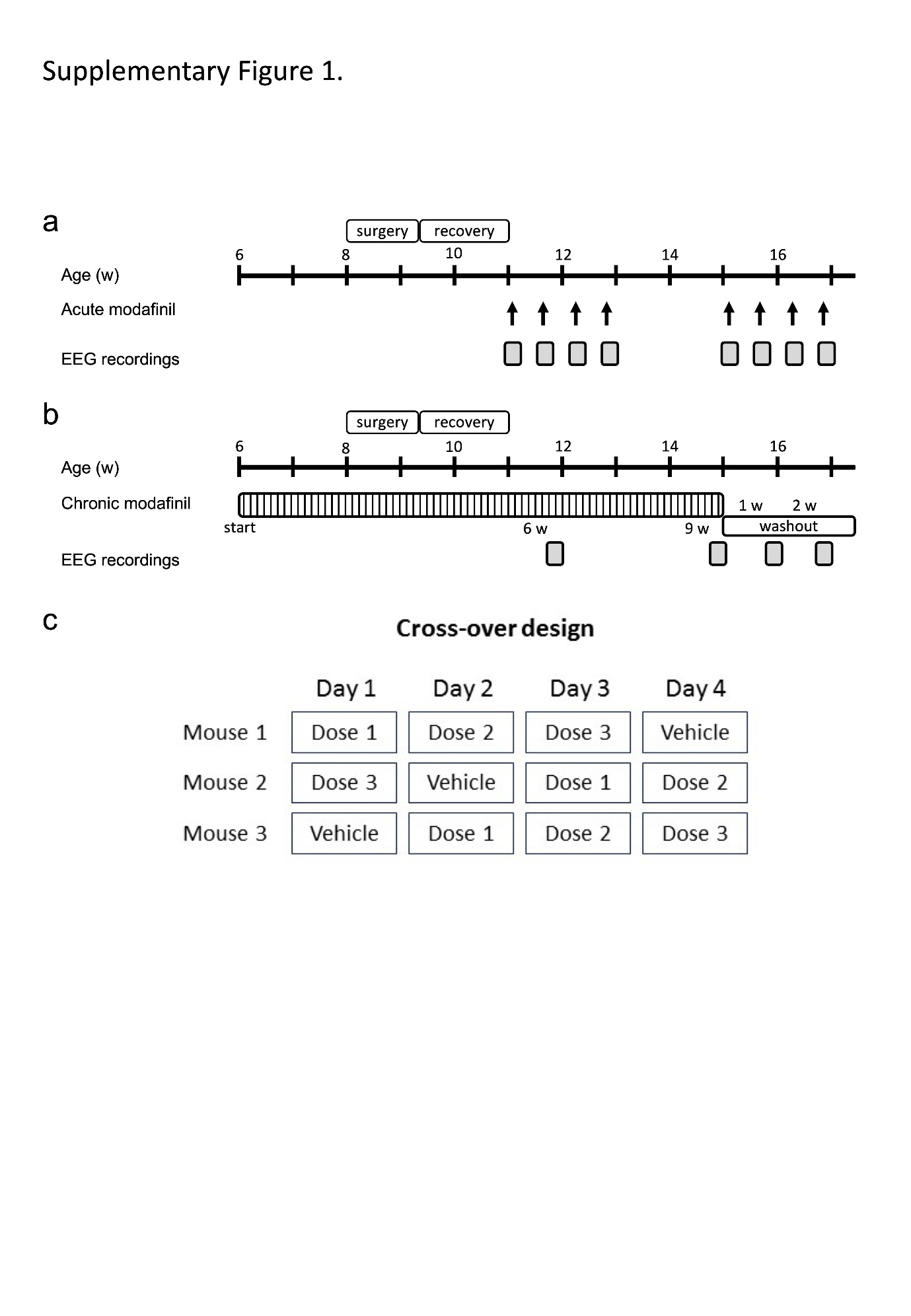
**

**Fig. S1 Timeline of modafinil treatment and electroencephalography (EEG) recordings on R6/2 mice.** For the acute treatment regime (a) mice were treated 4 times each (black arrows) with 3 doses of modafinil (25, 50 and 100 mg/kg) or vehicle in a crossover design with 3 days washout periods between injections at both 11-13 and 15-17 weeks of age. The treatments were given at the beginning of active phase (11am) and was followed by 24h EEG recording (blocks in a). In the chronic study (b), R6/2 mice were treated with 64 mg/kg modafinil or vehicle, starting at 6 weeks of age (shown by striped box). EEG recordings were made (starting at the beginning of active phase at 11am) for 24h at 4 time points, after 6 and 9 weeks after treatment and 1 and 2 weeks after the washout


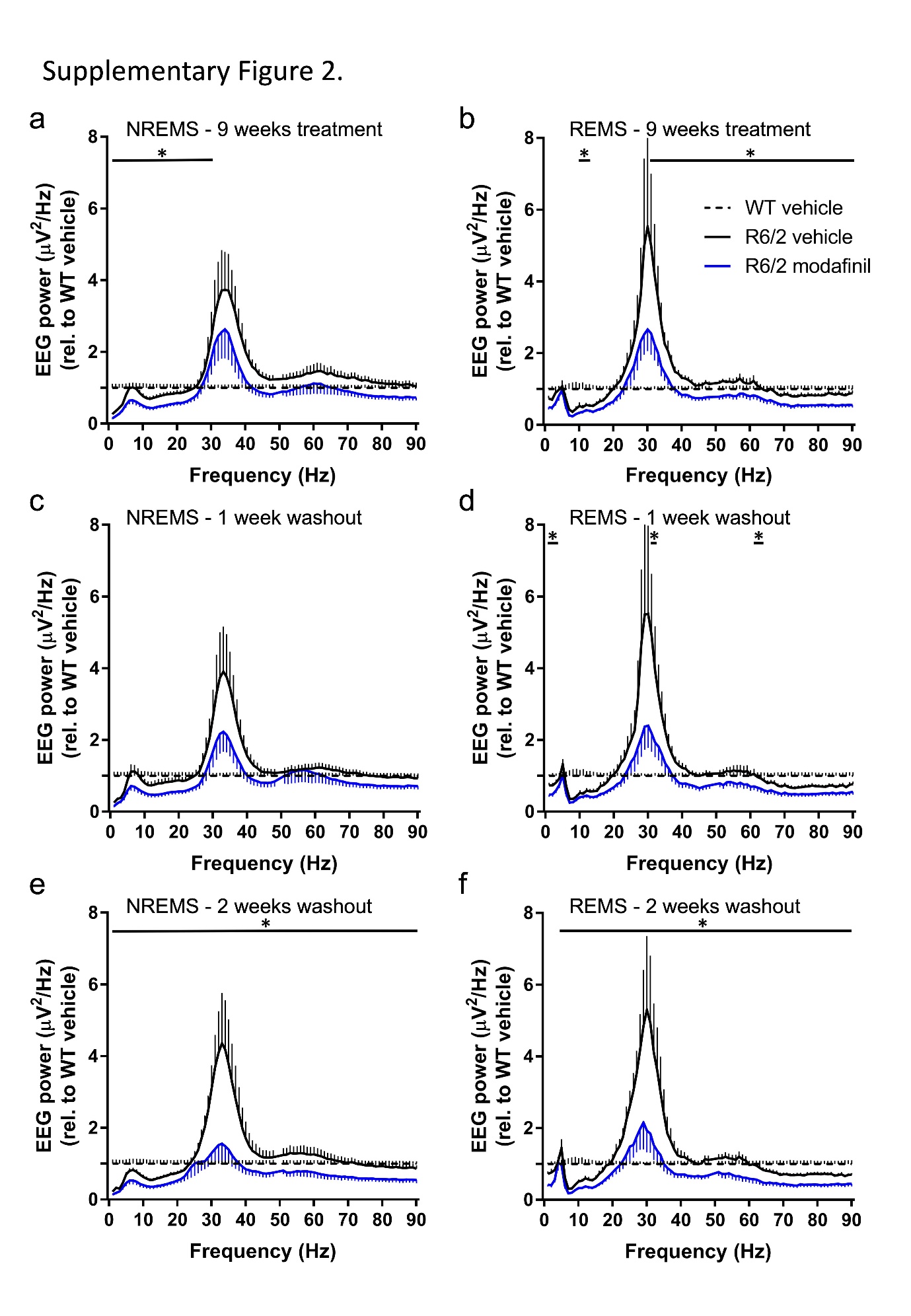


**Fig. S2 The effect of chronic modafinil treatment on the quantitative electroencephalography (EEG) in R6/2 mice measured during sleep.** EEG spectra of the vehicle- (solid black line) or modafinil- (solid blue line) treated groups in non-rapid eye movement sleep (NREMS, a, c and e) and rapid eye movement sleep (REMS, b, d and f) during the first 2h of the passive phase after 9 weeks of treatment (a and b, respectively) and following 1 (c and d, respectively) or 2 weeks of washout (e and f, respectively). Data were normalized to the WT vehicle group (dashed line). Data are presented as mean ± SEM in 1 Hz bins. Changes in the qEEG spectra following chronic modafinil treatment was quantified across the spectrum in standard frequency ranges (δ [1-4 Hz], θ [5-9 Hz], α [10-14 Hz], β [15-30 Hz], γ1 [31-60 Hz] and γ2 [61-90 Hz]). Statistically significant differences (two-way ANOVA and Bonferroni *post hoc* comparisons, * p < 0.05) are shown by black bars (modafinil vs. vehicle treatments) above the graphs

**
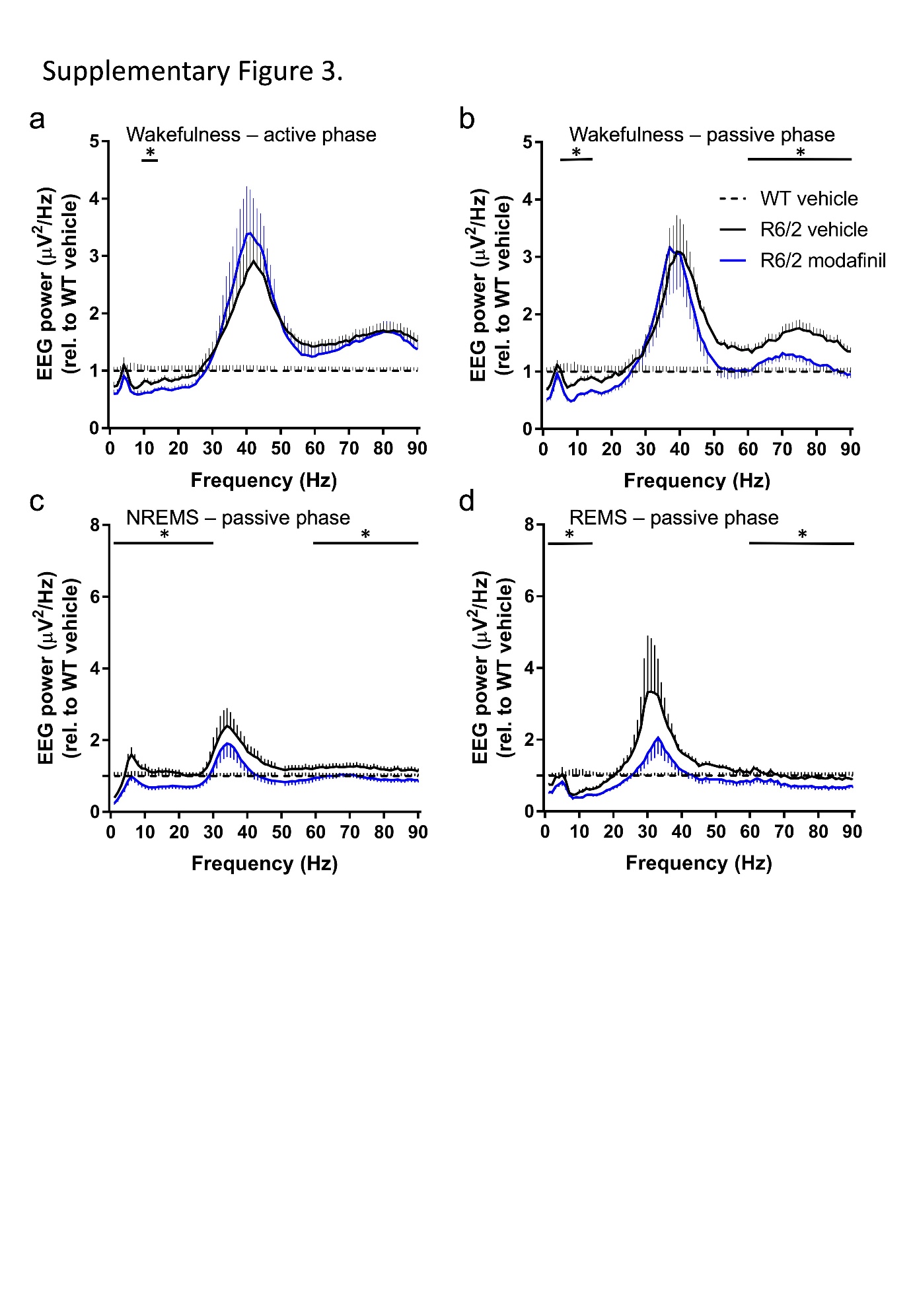
**

**Fig. S3 The effect of chronic modafinil treatment on the quantitative electroencephalography (EEG) in R6/2 mice measured during wakefulness and sleep after 6 weeks of treatment.** EEG spectra of the vehicle- (solid black line) or modafinil- (solid blue line) treated groups in wakefulness during the first 2h of the active phase or passive phase (a and b, respectively), as well as in non-rapid eye movement sleep (NREMS, c) and rapid eye movement sleep (REMS, d) in the first 2h of the passive phase. Data were normalized to the WT vehicle group (dashed line). Data are presented as mean ± SEM in 1 Hz bins. Changes in the qEEG spectra following chronic modafinil treatment was quantified across the spectrum in standard frequency ranges (δ [1-4 Hz], θ [5-9 Hz], α [10-14 Hz], β [15-30 Hz], γ1 [31-60 Hz] and γ2 [61-90 Hz]). Statistically significant differences (two-way ANOVA and Bonferroni *post hoc* comparisons, * p < 0.05) are shown by black bars (modafinil vs. vehicle treatments) above the graphs
