# Supplementary material for: Wake-Promoting and EEG Spectral Effects of Modafinil After Acute or Chronic Administration in the R6/2 Mouse Model of Huntington’s Disease
Source: Neurotherapeutics. 2020 Apr 15;17(3):1075–86. doi: 10.1007/s13311-020-00849-y (PMC7609772; doi:10.1007/s13311-020-00849-y)
Supplement: Supplementary file 8 — (DOCX 47.6 KB) [file 13311_2020_849_MOESM5_ESM.docx]

**Supplementary information**

**Wake-promoting and EEG spectral effects of modafinil after acute or chronic administration in the R6/2 mouse model of Huntington’s disease**

Szilvia Vas PhD^*^, Jackie M Casey^*^, Will T Schneider PhD^*^, Lajos Kalmar PhD† and A Jennifer Morton, PhD, ScD^*‡^

^*^Department of Physiology, Development and Neuroscience, University of Cambridge, Downing Street, Cambridge, CB2 3DY, UK, †Department of Veterinary Medicine, University of Cambridge, Madingley Road, Cambridge, CB3 0ES, UK

^‡^Author for correspondence

A. Jennifer Morton,

Department of Physiology, Development and Neuroscience,

University of Cambridge,

Downing Street,

Cambridge CB2 3DY,

United Kingdom

Phone: +44 1223 334057

Fax: +44 1223 333840

E-mail: ajm41@cam.ac.uk

**Supplementary tables**

**Table S1 The effect of acute modafinil treatment on sleep-wake pattern of R6/2 and wildtype mice during the passive phase**

|  |  | Vigilance state parameters | | | | | | | | |
| --- | --- | --- | --- | --- | --- | --- | --- | --- | --- | --- |
|  |  | Wake | | | NREMS | | | REMS | | |
| Genotype (age) | Treatment | Total time  (min) | Number of bouts | Mean bout duration (min) | Total time (min) | Number of bouts | Mean bout duration (min) | Total time (min) | Number of bouts | Mean bout duration (min) |
| Wildtype | Vehicle | 167.8 ± 7.7 | 96.6 ± 11.1 | 2.0 ± 0.3 | 478.7 ± 6.3 | 95.9 ± 4.9 | 5.2 ± 0.3 | 73.5 ± 2.9 | 73.9 ± 4.4 | 1.1 ± 0.0 |
|  | Modafinil | 164.2 ± 12.4 | 100.6 ± 10.0 | 1.7 ± 0.2 | 483.9 ± 10.4 | 100.3 ± 6.5 | 5.1 ± 0.4 | 71.9 ± 3.9 | 68.3 ± 5.3 | 1.1 ± 0.0 |
| R6/2  (12w) | Vehicle | 199.3 ± 15.3 | 92.4 ± 7.9 | 2.3 ± 0.2 | 430.3 ± 15.1 | 113.7 ± 4.6 | 3.9 ± 0.2 | 90.4 ± 2.0 | 85.7 ± 4.8 | 1.1 ± 0.1 |
|  | Modafinil | 164.1 ± 8.0* | 92.8 ± 8.9 | 1.9 ± 0.2 | 458 ± 7.7* | 107.8 ± 6.6 | 4.5 ± 0.3 | 96.2 ± 3.9 | 85.8 ± 6.7 | 1.2 ± 0.1 |
| R6/2  (16w) | Vehicle | 188.1 ± 9.1 | 92.1 ± 7.9 | 2.1 ± 0.1 | 446.6 ± 8.6 | 126.6 ± 9.3 | 3.8 ± 0.3 | 85.2 ± 4.5 | 83.8 ± 6.5 | 1.1 ± 0.1 |
|  | Modafinil | 180.9 ± 10.8 | 81.3 ± 7.0 | 2.4 ± 0.3 | 448 ± 8.2 | 116.6 ± 9.6 | 4.2 ± 0.4 | 91.2 ± 4.1 | 88.2 ± 8.7 | 1.1 ± 0.1 |

Data are presented as mean ± SEM. *: *P* < 0.05, modafinil 100 mg/kg *vs* the relevant vehicle-treated group.

**Table S2 Statistical analysis of the quantitative EEG spectra during wakefulness in the first 2h of the active phase in vehicle treated R6/2 mice at 12 and 16 weeks of age compared to wildtype mice**

| **Age** | **EEG Frequency range** | **Results of two-way ANOVA**  **(Genotype)** | | **Results of two-way ANOVA**  **(Interaction)** | | **Frequency ranges**  **with differences** |
| --- | --- | --- | --- | --- | --- | --- |
|  |  | ***F* value** | ***P* value** | ***F* value** | ***P* value** | **(Hz)** |
| **12 weeks** | | |  |  |  |  |
|  | delta | F_(1,16)_ = 0.39 | 0.5374 | **F_(3,48)_ = 5.30** | **0.0031** | - |
|  | theta | F_(1,16)_ = 0.00 | 0.9740 | F_(4,64)_ = 0.28 | 0.8892 | - |
|  | alpha | F_(1,16)_ = 2.53 | 0.1313 | F_(4,64)_ = 0.84 | 0.5031 | - |
|  | beta | F_(1,16)_ = 0.09 | 0.7578 | **F_(15,240)_ = 15.23** | **<0.0001** | - |
|  | low gamma | **F_(1,16)_ = 24.08** | **0.0002** | **F_(27,432)_ = 12.76** | **<0.0001** | 34-48 |
|  | high gamma | **F_(1,16)_ = 9.19** | **0.0079** | **F_(29,464)_ = 2.95** | **<0.0001** | 61-67 |
| **16 weeks** | | |  |  |  |  |
|  | delta | F_(1,16)_ = 1.29 | 0.2726 | **F_(3,48)_ = 15.03** | **<0.0001** | - |
|  | theta | F_(1,16)_ = 1.03 | 0.3245 | F_(4,64)_ = 1.36 | 0.2592 | - |
|  | alpha | **F_(1,16)_ = 8.16** | **0.0114** | **F_(4,64)_ = 2.82** | **0.0322** | 10-12 |
|  | beta | F_(1,16)_ = 0.06 | 0.8075 | **F_(15,240)_ = 14.46** | **<0.0001** | 29-30 |
|  | low gamma | **F_(1,16)_ = 18.74** | **0.0005** | **F_(27,432_ = 11.89** | **<0.0001** | 31-43 |
|  | high gamma | **F_(1,16)_ = 6.67** | **0.0200** | **F_(29,464)_ = 5.87** | **<0.0001** | 61-69 |

*P* and *F* values are indicated for the effect of genotype and the interaction between the two factors. *P* and *F* values of significant ANOVAs are shown in bold. Bonferroni *post hoc* comparison test was used to identify frequencies with significant (*P* < 0.05) differences.

**Table S3 Statistical analysis of the effect of acute modafinil on the quantitative EEG spectra of wakefulness measured during the first 2h of active phase in wildtype and R6/2 mice**

| **Genotype/**  **Age of mice** | **EEG Frequency**  **range** | **Direction of change** | **Results of two-way ANOVA**  **(Treatment)** | | **Results of two-way ANOVA**  **(Interaction)** | | **Frequency ranges with differences**  **(Hz)** |
| --- | --- | --- | --- | --- | --- | --- | --- |
|  |  |  | ***F* value** | ***P* value** | ***F* value** | ***P* value** |  |
| **Wild-type**  **(12 weeks)** | |  |  |  |  |  |  |
|  | delta (1-4 Hz) | **↓** | **F_(1,8)_ = 20.55** | **0.0019** | **F_(3,24)_ = 69.64** | **<0.0001** | 1, 3-4 |
|  | theta (5-9 Hz) | **↓** | F_(1,8)_ = 4.06 | 0.0787 | **F_(4,32)_ = 12.40** | **<0.0001** | 5-6 |
|  | alpha (10-14 Hz) | **↓** | **F_(1,8)_ = 14.54** | **0.0051** | F_(4,32)_ = 2.38 | 0.0721 | 11-14 |
|  | beta (15-30 Hz) | **↓↑** | F_(1,8)_ = 3.74 | 0.0893 | **F_(15,120)_ = 18.22** | **<0.0001** | 15-20, 29-30 |
|  | low gamma (31-60 Hz) | **↑** | **F_(1,8)_ = 12.13** | **0.0083** | **F_(27,216)_ = 7.39** | **<0.0001** | 31-48 |
|  | high gamma (61-90 Hz) | **↑** | **F_(1,8)_ = 14.74** | **0.0050** | **F_(29,232)_ = 2.47** | **0.0001** | 61-90 |
| **R6/2**  **(12 weeks)** | |  | |  |  |  |  |
|  | delta (1-4 Hz) | **↓** | **F_(1,8)_ = 18.58** | **0.0026** | **F_(3,24)_ = 26.17** | **<0.0001** | 3-4 |
|  | theta (5-9 Hz) | - | F_(1,8)_ = 0.71 | 0.4239 | **F_(4,32)_ = 3.99** | **0.0097** | 5 |
|  | alpha (10-14 Hz) | - | F_(1,8)_ = 3.69 | 0.0907 | F_(4,32)_ = 0.81 | 0.5275 | - |
|  | beta (15-30 Hz) | **↑** | F_(1,8)_ = 4.92 | 0.0573 | **F_(15,120)_ = 8.62** | **<0.0001** | 25-30 |
|  | low gamma (31-60 Hz) | **↑** | **F_(1,8)_ = 35.27** | **0.0003** | **F_(27,216)_ = 10.90** | **<0.0001** | 31-43 |
|  | high gamma (61-90 Hz) | **↑** | **F_(1,8)_ = 10.31** | **0.0124** | F_(29,232)_ = 1.35 | 0.1165 | 62-89 |
| **R6/2**  **(16 weeks)** | |  | |  |  |  |  |
|  | delta (1-4 Hz) | - | F_(1,8)_ = 0.22 | 0.6512 | **F_(3,24)_ = 4.69** | **0.0102** | 4 |
|  | theta (5-9 Hz) | - | F_(1,8)_ = 0.17 | 0.6942 | **F_(4,32)_ = 4.94** | **0.0032** | 6 |
|  | alpha (10-14 Hz) | **↓** | F_(1,8)_ = 4.25 | 0.0732 | **F_(4,32)_ = 5.68** | **0.0014** | 10-11 |
|  | beta (15-30 Hz) | **↓** | F_(1,8)_ = 0.06 | 0.8116 | **F_(15,120)_ = 3.99** | **<0.0001** | 29-30 |
|  | low gamma (31-60 Hz) | - | F_(1,8)_ = 0.28 | 0.6113 | F_(27,216)_ = 1.13 | 0.3130 | - |
|  | high gamma (61-90 Hz) | **↑** | **F_(1,8)_ = 5.75** | **0.0432** | **F_(29,232)_ = 3.01** | **<0.0001** | 61-82 |

*P* and *F* values are indicated for the effect of the treatment (100 mg/kg modafinil or vehicle) and the interaction between the two factors. *P* and *F* values of significant ANOVAs are shown in bold. Bonferroni *post hoc* comparison test was used to identify frequencies with significant (*P* < 0.05) differences.

**Table S4 EEG power in theta band (5-9 Hz) after acute treatment with modafinil (100 mg/kg)**

|  |  |  | **EEG power (μV^2^)** | | | | |
| --- | --- | --- | --- | --- | --- | --- | --- |
| **Genotype** | **Age**  **(weeks)** | **Treatment** | **Frequency bins of the theta range** | | | | |
|  |  |  | **5 Hz** | **6 Hz** | **7 Hz** | **8 Hz** | **9 Hz** |
| Wildtype | 12 | Vehicle | **182.6 ± 19.5** | **180.5 ± 25.8** | 156.9 ± 21.6 | 116.6 ± 14.2 | 82.9 ± 9.5 |
|  |  | Modafinil | 102.0 ± 9.6 | 140.7 ± 24.0 | **169.3 ± 35.3** | 149.9 ± 26.4 | 98.6 ± 15.0 |
| R6/2 | 12 | Vehicle | **175.6 ± 14.2** | **177.2 ± 20.6** | 163.8 ± 23.1 | 122.1 ± 19.2 | 76.8 ± 11.3 |
|  |  | Modafinil | 137.4 ± 17.0 | 164.8 ± 20.3 | **177.8 ± 26.1** | 136.0 ± 29.7 | 77.4 ± 16.7 |
|  | 16 | Vehicle | **182.2 ± 16.6** | 166.9 ± 19.0 | 125.3 ± 16.3 | 82.5 ± 10.0 | 56.3 ± 4.9 |
|  |  | Modafinil | 174.1 ± 20.7 | **184.0 ± 22.3** | 138.6 ± 20.6 | 78.1 ± 11.2 | 49.7 ± 5.2 |

Bold: 1 Hz bins of the theta frequency range where the highest theta power was measured.

**Table S5 Quantification of sleep-wake pattern of R6/2 mice treated with chronic modafinil measured during the 12h of passive phase**

| **Treatment &**  **duration** | **Vehicle** | **Modafinil** | **Vehicle** | **Modafinil** | **Vehicle** | **Modafinil** | **Vehicle** | **Modafinil** |
| --- | --- | --- | --- | --- | --- | --- | --- | --- |
|  | **6 weeks** | | **9 weeks** | | **1 week washout** | | **2 weeks washout** | |
| **Age of mice** | **12 weeks** | | **15 weeks** | | **16 weeks** | | **17 weeks** | |
| **Vigilance states** |  |  |  |  |  |  |  |  |
| **Wake** |  |  |  |  |  |  |  |  |
| Total time (min) | 217.9 ± 15 | 229.5 ± 15 | 235.9 ± 11 | 236.2 ± 15 | 243.6 ± 20 | 248.3 ± 29 | 257.2 ± 16 | 271 ± 24 |
| Number of bouts | 112.5 ± 8 | 120.2 ± 6 | 121.5 ± 6 | 124 ± 9 | 127.3 ± 8 | 114.3 ± 9 | 131.9 ± 18 | 138.8 ± 15 |
| Mean bout duration (min) | 2.1 ± 0 | 2.0 ± 0 | 2.0 ± 0 | 2.0 ± 0 | 2.0 ± 0 | 2.3 ± 0 | 2.2 ± 0 | 2.1 ± 0 |
| **NREMS** |  |  |  |  |  |  |  |  |
| Total time (min) | 446 ± 16 | 434.9 ± 18 | 433.9 ± 15 | 428.8 ± 16 | 416.6 ± 26 | 380.3 ± 26 | 416.1 ± 14 | 387.8 ± 24 |
| Number of bouts | 114.8 ± 8 | 110.2 ± 6 | 123.3 ± 6 | 122.3 ± 10 | 135.8 ± 9 | 146.9 ± 8 | 132.6 ± 16 | 141.6 ± 11 |
| Mean bout duration (min) | 4.1 ± 0 | 4.2 ± 0 | 3.7 ± 0 | 3.9 ± 0 | 3.3 ± 3 | 2.8 ± 0 | 3.6 ± 0 | 3.0 ± 0 |
| **REMS** |  |  |  |  |  |  |  |  |
| Total time (min) | 56.1 ± 7 | 55.7 ± 7 | 50.2 ± 5 | 54.9 ± 7 | 59.8 ± 9 | 91.4 ± 16 | 46.7 ± 6 | 61.3 ± 9 |
| Number of bouts | 59.8 ± 6 | 67.9 ± 9 | 63.8 ± 10 | 70.5 ± 14 | 79.3 ± 14 | 120.2 ± 17 | 58.6 ± 15 | 98.6 ± 14 |
| Mean bout duration (min) | 1.1 ± 0 | 1.1 ± 0 | 1.1 ± 0 | 1.1 ± 0 | 1.2 ± 0 | 1.2 ± 0 | 1.1 ± 0 | 1.2 ± 0** |

Data are presented as mean ± SEM. **: *P* < 0.01, modafinil 64 mg/kg (n = 10) *vs* the relevant vehicle-treated group (n = 8).

**Table S6 Statistical analysis of the effect of 9 weeks chronic modafinil treatment on the quantitative EEG spectra of wakefulness measured during the first 2h of active and passive phase in R6/2 mice**

| **9 weeks of treatment** | |  |  | |  | |  |
| --- | --- | --- | --- | --- | --- | --- | --- |
| **Phase**  **tested** | **EEG Frequency**  **range** | **Direction of change** | **Results of two-way ANOVA**  **(Treatment)** | | **Results of two-way**  **ANOVA (Interaction)** | | **Frequency ranges with differences**  **(Hz)** |
|  |  |  | ***F* value** | ***P* value** | ***F* value** | ***P* value** |  |
| **Active** | |  |  |  |  |  |  |
|  | delta (1-4 Hz) | **-** | F_(1,16)_ = 1.37 | 0.2585 | F_(3,48)_ = 1.09 | 0.1419 | - |
|  | theta (5-9 Hz) | **↓** | **F_(1,16)_ = 4.65** | **0.0466** | F_(4,64)_ = 0.94 | 0.4466 | - |
|  | alpha (10-14 Hz) | **-** | F_(1,16)_ = 2.55 | 0.1302 | **F_(4,64)_ = 6.98** | **0.0001** | 5 |
|  | beta (15-30 Hz) | **-** | F_(1,16)_ = 1.47 | 0.2426 | F_(15,240)_ = 0.27 | 0.9974 | 10 |
|  | low gamma (31-60 Hz) | **-** | F_(1,16)_ = 0.06 | 0.8041 | F_(27,432)_ = 0.21 | >0.9999 | - |
|  | high gamma (61-90 Hz) | **-** | F_(1,16)_ = 0.49 | 0.4949 | F_(29,464)_ = 1.45 | 0.0640 | - |
| **Passive** | |  | |  |  |  |  |
|  | delta (1-4 Hz) | **-** | F_(1,16)_ = 1.54 | 0.2331 | F_(3,48)_ = 0.35 | 0.7866 | - |
|  | theta (5-9 Hz) | **↓** | **F_(1,16)_ = 6.24** | **0.0238** | F_(4,64)_ = 1.50 | 0.2121 | 6 |
|  | alpha (10-14 Hz) | **↓** | **F_(1,16)_ = 4.72** | **0.0452** | F_(4,64)_ = 1.51 | 0.2105 | 10 |
|  | beta (15-30 Hz) | **-** | F_(1,16)_ = 1.91 | 0.1858 | F_(15,240)_ = 0.21 | 0.9994 | - |
|  | low gamma (31-60 Hz) | **-** | F_(1,16)_ = 1.18 | 0.2939 | F_(27,432)_ = 0.72 | 0.8446 | - |
|  | high gamma (61-90 Hz) | **↓** | **F_(1,16)_ = 5.04** | **0.0393** | **F_(29,464)_ = 2.24** | **0.0003** | 62, 64 |

*P* and *F* values are indicated for the effect of the treatment (100 mg/kg modafinil or vehicle) and the interaction between the two factors. *P* and *F* values of significant ANOVAs are shown in bold. Bonferroni *post hoc* comparison test was used to identify frequencies with significant (*P*<0.05) differences.

**Table S7 Statistical analysis of the effect of chronic modafinil treatment on the quantitative EEG spectra of wakefulness measured during the first 2h of active and passive phase in R6/2 mice after one week of washout**

| **1 week of washout** | |  |  | |  | |  |
| --- | --- | --- | --- | --- | --- | --- | --- |
| **Phase**  **tested** | **EEG Frequency**  **range** | **Direction of change** | **Results of two-way ANOVA**  **(Treatment)** | | **Results of two-way ANOVA**  **(Interaction)** | | **Frequency ranges with differences**  **(Hz)** |
|  |  |  | ***F* value** | ***P* value** | ***F* value** | ***P* value** |  |
| **Active** | |  |  |  |  |  |  |
|  | delta (1-4 Hz) | **-** | F_(1,16)_ = 1.58 | 0.2264 | F_(3,48)_ = 0.81 | 0.4949 | - |
|  | theta (5-9 Hz) | **↓** | F_(1,16)_ = 2.14 | 0.1631 | F_(4,64)_ = 0.49 | 0.7399 | - |
|  | alpha (10-14 Hz) | **-** | F_(1,16)_ = 2.62 | 0.1250 | F_(4,64)_ = 0.32 | 0.8642 | - |
|  | beta (15-30 Hz) | **-** | F_(1,16)_ = 3.34 | 0.0865 | F_(15,240)_ = 0.03 | >0.9999 | - |
|  | low gamma (31-60 Hz) | **-** | F_(1,16)_ = 2.36 | 0.1438 | **F_(27,432)_ = 1.73** | **0.0140** | 39 |
|  | high gamma (61-90 Hz) | **-** | F_(1,16)_ = 1.17 | 0.2950 | F_(29,464)_ = 0.43 | 0.9961 | - |
| **Passive** | |  | |  |  |  |  |
|  | delta (1-4 Hz) | **-** | F_(1,16)_ = 1.61 | 0.2226 | F_(3,48)_ = 0.71 | 0.5530 | - |
|  | theta (5-9 Hz) | **↓** | **F_(1,16)_ = 4.89** | **0.0419** | F_(4,64)_ = 1.64 | 0.1759 | 6 |
|  | alpha (10-14 Hz) | **↓** | F_(1,16)_ = 3.84 | 0.0677 | F_(4,64)_ = 1.21 | 0.3136 | - |
|  | beta (15-30 Hz) | **-** | F_(1,16)_ = 0.95 | 0.3436 | F_(15,240)_ = 0.61 | 0.8648 | - |
|  | low gamma (31-60 Hz) | **-** | F_(1,16)_ = 1.89 | 0.1886 | F_(27,432)_ = 1.32 | 0.1313 | - |
|  | high gamma (61-90 Hz) | **↓** | F_(1,16)_ = 2.66 | 0.1227 | **F_(29,464)_ = 1.51** | **0.0452** | - |

*P* and *F* values are indicated for the effect of the treatment (100 mg/kg modafinil or vehicle) and the interaction between the two factors. *P* and *F* values of significant ANOVAs are shown in bold. Bonferroni *post hoc* comparison test was used to identify frequencies with significant (*P* < 0.05) differences.

**Table S8 Statistical analysis of the effect of chronic modafinil on the quantitative EEG spectra of wakefulness measured during the first 2h of active and passive phase in R6/2 mice after two weeks of washout**

| **2 weeks of washout** | |  |  | |  | |  |
| --- | --- | --- | --- | --- | --- | --- | --- |
| **Phase**  **tested** | **EEG Frequency**  **range** | **Direction of change** | **Results of two-way ANOVA**  **(Treatment)** | | **Results of two-way ANOVA**  **(Interaction)** | | **Frequency ranges with differences**  **(Hz)** |
|  |  |  | ***F* value** | ***P* value** | ***F* value** | ***P* value** |  |
| **Active** | |  |  |  |  |  |  |
|  | delta (1-4 Hz) | **-** | F_(1,16)_ = 3.09 | 0.0980 | F_(3,48)_ = 1.58 | 0.2056 | - |
|  | theta (5-9 Hz) | **↓** | **F_(1,16)_ = 5.79** | **0.0286** | F_(4,64)_ = 1.62 | 0.1814 | 5 |
|  | alpha (10-14 Hz) | **↓** | **F_(1,16)_ = 6.55** | **0.0210** | **F_(4,64)_ = 6.08** | **0.0003** | 10 |
|  | beta (15-30 Hz) | **-** | F_(1,16)_ = 1.33 | 0.2666 | F_(15,240)_ = 0.65 | 0.8350 | - |
|  | low gamma (31-60 Hz) | **↓** | F_(1,16)_ = 3.15 | 0.0949 | **F_(27,432)_ = 2.45** | **<0.0001** | 38-40 |
|  | high gamma (61-90 Hz) | **↓** | F_(1,16)_ = 3.90 | 0.0659 | **F_(29,464)_ = 2.78** | **<0.0001** | 61-64 |
| **Passive** | |  | |  |  |  |  |
|  | delta (1-4 Hz) | - | F_(1,16)_ = 2.55 | 0.1300 | F_(3,48)_ = 2.25 | 0.0948 | - |
|  | theta (5-9 Hz) | **↓** | **F_(1,16)_ = 9.51** | **0.0071** | **F_(4,64)_ = 5.68** | **0.0006** | 5-7 |
|  | alpha (10-14 Hz) | **↓** | **F_(1,16)_ = 6.05** | **0.0257** | **F_(4,64)_ = 3.48** | **0.0123** | 10 |
|  | beta (15-30 Hz) | - | F_(1,16)_ = 1.59 | 0.2258 | F_(15,240)_ = 0.39 | 0.9805 | - |
|  | low gamma (31-60 Hz) | **↓** | **F_(1,16)_ = 7.38** | **0.0153** | **F_(27,432)_ = 4.05** | **<0.0001** | 33-39 |
|  | high gamma (61-90 Hz) | **↓** | **F_(1,16)_ = 4.96** | **0.0407** | **F_(29,464)_ = 3.60** | **<0.0001** | 61-64 |

*P* and *F* values are indicated for the effect of the treatment (100 mg/kg modafinil or vehicle) and the interaction between the two factors. *P* and *F* values of significant ANOVAs are shown in bold. Bonferroni *post hoc* comparison test was used to identify frequencies with significant (*P* < 0.05) differences.

**Table S9 Statistical analysis of the effect of chronic modafinil on the quantitative EEG spectra of NREMS and REMS measured during the first 2h of passive phase in R6/2 mice after 9 weeks of treatment**

| **9 weeks of treatment** | |  |  | |  | |  |
| --- | --- | --- | --- | --- | --- | --- | --- |
| **Vigilance state** | **EEG Frequency**  **range** | **Direction of change** | **Results of two-way ANOVA**  **(Treatment)** | | **Results of two-way ANOVA**  **(Interaction)** | | **Frequency ranges with differences**  **(Hz)** |
|  |  |  | ***F* value** | ***P* value** | ***F* value** | ***P* value** |  |
| **NREMS** | |  |  |  |  |  |  |
|  | delta (1-4 Hz) | **↓** | **F_(1,16)_ = 6.87** | **0.0185** | F_(3,48)_ = 2.11 | 0.1116 | 2-3 |
|  | theta (5-9 Hz) | **↓** | **F_(1,16)_ = 5.99** | **0.0263** | **F_(4,64)_ = 3.50** | **0.0120** | 5-6 |
|  | alpha (10-14 Hz) | **↓** | **F_(1,16)_ = 7.50** | **0.0146** | **F_(4,64)_ = 3.50** | **0.0120** | 10-11 |
|  | beta (15-30 Hz) | **↓** | **F_(1,16)_ = 4.70** | **0.0455** | F_(15,240)_ = 1.47 | 0.1165 | 15-16 |
|  | low gamma (31-60 Hz) | **-** | F_(1,16)_ = 1.53 | 0.2341 | F_(27,432)_ = 0.67 | 0.8962 | - |
|  | high gamma (61-90 Hz) | **-** | F_(1,16)_ = 3.49 | 0.0803 | F_(29,464)_ = 0.51 | 0.9840 | - |
| **REMS** | |  | |  |  |  |  |
|  | delta (1-4 Hz) | **-** | F_(1,16)_ = 4.33 | 0.0540 | F_(3,48)_ = 0.50 | 0.6876 | - |
|  | theta (5-9 Hz) | **-** | F_(1,16)_ = 2.25 | 0.1534 | F_(4,64)_ = 1.41 | 0.2406 | - |
|  | alpha (10-14 Hz) | **↓** | **F_(1,16)_ = 4.54** | **0.0489** | F_(4,64)_ = 1.32 | 0.2707 | 10 |
|  | beta (15-30 Hz) | **-** | F_(1,16)_ = 1.88 | 0.9137 | F_(15,240)_ = 0.91 | 0.5495 | - |
|  | low gamma (31-60 Hz) | **↓** | **F_(1,16)_ = 5.11** | **0.0381** | **F_(27,432)_ = 1.58** | **0.0348** | 31-32 |
|  | high gamma (61-90 Hz) | **↓** | **F_(1,16)_ = 6.99** | **0.0177** | **F_(29,464)_ = 2.26** | **0.0002** | 61-64 |

*P* and *F* values are indicated for the effect of the treatment (100 mg/kg modafinil or vehicle) and the interaction between the two factors. *P* and *F* values of significant ANOVAs are shown in bold. Bonferroni *post hoc* comparison test was used to identify frequencies with significant (*P* < 0.05) differences.

**Table S10 Statistical analysis of the effect of chronic modafinil on the quantitative EEG spectra of NREMS and REMS measured during the first 2h of passive phase in R6/2 mice after 1 week of washout**

| **1 week washout** | |  |  | |  | |  |
| --- | --- | --- | --- | --- | --- | --- | --- |
| **Vigilance state** | **EEG Frequency**  **range** | **Direction of change** | **Results of two-way ANOVA**  **(Treatment)** | | **Results of two-way ANOVA**  **(Interaction)** | | **Frequency ranges with differences**  **(Hz)** |
|  |  |  | ***F* value** | ***P* value** | ***F* value** | ***P* value** |  |
| **NREMS** | |  |  |  |  |  |  |
|  | delta (1-4 Hz) | **-** | F_(1,16)_ = 2.65 | 0.1233 | F_(3,48)_ = 0.56 | 0.6455 | - |
|  | theta (5-9 Hz) | **-** | F_(1,16)_ = 3.40 | 0.0838 | F_(4,64)_ = 1.60 | 0.1867 | - |
|  | alpha (10-14 Hz) | **-** | F_(1,16)_ = 4.06 | 0.0612 | F_(4,64)_ = 2.02 | 0.1029 | - |
|  | beta (15-30 Hz) | **-** | F_(1,16)_ = 4.45 | 0.0509 | F_(15,240)_ = 0.97 | 0.4844 | - |
|  | low gamma (31-60 Hz) | **-** | F_(1,16)_ = 1.88 | 0.1893 | **F_(27,432)_ = 1.68** | **0.0195** | - |
|  | high gamma (61-90 Hz) | **-** | F_(1,16)_ = 2.26 | 0.1521 | F_(29,464)_ = 0.33 | 0.9997 | - |
| **REMS** | |  | |  |  |  |  |
|  | delta (1-4 Hz) | **-** | **F_(1,16)_ = 4.60** | **0.0478** | F_(3,48)_ = 0.47 | 0.7035 | - |
|  | theta (5-9 Hz) | **-** | F_(1,16)_ = 2.85 | 0.1110 | F_(4,64)_ = 2.24 | 0.0747 | - |
|  | alpha (10-14 Hz) | **-** | F_(1,16)_ = 2.25 | 0.1531 | F_(4,64)_ = 0.42 | 0.7954 | - |
|  | beta (15-30 Hz) | **-** | F_(1,16)_ = 2.29 | 0.1495 | F_(15,240)_ = 1.37 | 0.1619 | - |
|  | low gamma (31-60 Hz) | **↓** | F_(1,16)_ = 4.38 | 0.0526 | **F_(27,432)_ = 2.33** | **0.0002** | 31-32 |
|  | high gamma (61-90 Hz) | **↓** | F_(1,16)_ = 4.25 | 0.0559 | **F_(29,464)_ = 2.85** | **<0.0001** | 61-64 |

*P* and *F* values are indicated for the effect of the treatment (100 mg/kg modafinil or vehicle) and the interaction between the two factors. *P* and *F* values of significant ANOVAs are shown in bold. Bonferroni *post hoc* comparison test was used to identify frequencies with significant (*P* < 0.05) differences.

**Table S11 Statistical analysis of the effect of chronic modafinil on the quantitative EEG spectra of NREMS and REMS measured during the first 2h of passive phase in R6/2 mice after 2 weeks of washout**

| **2 weeks washout** | |  |  | |  | |  |
| --- | --- | --- | --- | --- | --- | --- | --- |
| **Vigilance state** | **EEG Frequency**  **range** | **Direction of change** | **Results of two-way ANOVA**  **(Treatment)** | | **Results of two-way ANOVA**  **(Interaction)** | | **Frequency ranges with differences**  **(Hz)** |
|  |  |  | ***F* value** | ***P* value** | ***F* value** | ***P* value** |  |
| **NREMS** | |  |  |  |  |  |  |
|  | delta (1-4 Hz) | **-** | **F_(1,16)_ = 4.93** | **0.0411** | F_(3,48)_ = 2.16 | 0.1050 | 2 |
|  | theta (5-9 Hz) | **↓** | **F_(1,16)_ = 5.18** | **0.0370** | F_(4,64)_ = 2.13 | 0.0868 | 5 |
|  | alpha (10-14 Hz) | **↓** | **F_(1,16)_ = 5.53** | **0.0318** | **F_(4,64)_ = 4.67** | **0.0023** | 10 |
|  | beta (15-30 Hz) | **↓** | **F_(1,16)_ = 5.54** | **0.0317** | **F_(15,240)_ = 2.36** | **0.0035** | 29-30 |
|  | low gamma (31-60 Hz) | **↓** | **F_(1,16)_ = 4.70** | **0.0456** | **F_(27,432)_ = 4.28** | **<0.0001** | 31-35 |
|  | high gamma (61-90 Hz) | **↓** | **F_(1,16)_ = 6.44** | **0.0220** | **F_(29,464)_ = 4.24** | **<0.0001** | 61-66 |
| **REMS** | |  | |  |  |  |  |
|  | delta (1-4 Hz) | **-** | F_(1,16)_ = 3.00 | 0.1027 | F_(3,48)_ = 1.07 | 0.3727 | - |
|  | theta (5-9 Hz) | **↓** | **F_(1,16)_ = 5.43** | **0.0332** | F_(4,64)_ = 2.04 | 0.0995 | 5-6 |
|  | alpha (10-14 Hz) | **↓** | **F_(1,16)_ = 8.92** | **0.0087** | **F_(4,64)_ = 2.53** | **0.0490** | 10-12 |
|  | beta (15-30 Hz) | **↓** | **F_(1,16)_ = 5.28** | **0.0354** | **F_(15,240)_ = 1.95** | **0.0195** | 29-30 |
|  | low gamma (31-60 Hz) | **↓** | **F_(1,16)_ = 5.71** | **0.0285** | **F_(27,432)_ = 2.89** | **<0.0001** | 31-33 |
|  | high gamma (61-90 Hz) | **↓** | **F_(1,16)_ = 8.69** | **0.0094** | **F_(29,464)_ = 5.58** | **<0.0001** | 61-67 |

*P* and *F* values are indicated for the effect of the treatment (100 mg/kg modafinil or vehicle) and the interaction between the two factors. *P* and *F* values of significant ANOVAs are shown in bold. Bonferroni *post hoc* comparison test was used to identify frequencies with significant (*P* < 0.05) differences.

**Table S12 Statistical analysis of the effect of chronic modafinil on the quantitative EEG spectra of wakefulness measured during the first 2h of active and passive phase in R6/2 mice after 6 weeks of treatment**

| **6 weeks of treatment** | | |  | | |  | | | |  | | |  |  |
| --- | --- | --- | --- | --- | --- | --- | --- | --- | --- | --- | --- | --- | --- | --- |
| **Vigilance state** | | **EEG Frequency**  **range** | **Direction of change** | | | **Results of two-way ANOVA**  **(Treatment)** | | | | **Results of two-way ANOVA**  **(Interaction)** | | | **Frequency ranges with differences**  **(Hz)** |  |
|  | |  |  | | | ***F* value** | | ***P* value** | | ***F* value** | | ***P* value** |  |  |
| **Wakefulness – active phase** | | |  | | |  | |  | |  | |  |  |  |
|  | delta (1-4 Hz) | | | **-** | F_(1,16)_ = 1.73 | | 0.2068 | | **F_(3,48)_ = 0.87** | | **0.0466** | | - | |
|  | theta (5-9 Hz) | | | **-** | F_(1,16)_ = 2.59 | | 0.1270 | | **F_(4,64)_ = 0.39** | | **0.0187** | | - | |
|  | alpha (10-14 Hz) | | | **↓** | **F_(1,16)_ = 6.46** | | **0.0218** | | **F_(4,64)_ = 8.84** | | **<0.0001** | | 10-11 | |
|  | beta (15-30 Hz) | | | **-** | F_(1,16)_ = 2.09 | | 0.1678 | | F_(15,240)_ = 0.52 | | 0.9287 | | 15-16 | |
|  | low gamma (31-60 Hz) | | | **-** | F_(1,16)_ = 0.15 | | 0.7059 | | F_(27,432)_ = 0.47 | | 0.9897 | | - | |
|  | high gamma (61-90 Hz) | | | **-** | F_(1,16)_ = 0.34 | | 0.5683 | | F_(29,464)_ = 0.52 | | 0.9828 | | - | |
| **Wakefulness – passive phase** | | | |  | | |  | |  | |  | |  | |
|  | delta (1-4 Hz) | | | **-** | F_(1,16)_ = 2.48 | | 0.1352 | | F_(3,48)_ = 0.14 | | 0.9366 | | - | |
|  | theta (5-9 Hz) | | | **↓** | **F_(1,16)_ = 11.89** | | **0.0033** | | F_(4,64)_ = 0.36 | | 0.8370 | | 5-6, 8 | |
|  | alpha (10-14 Hz) | | | **↓** | **F_(1,16)_ = 17.10** | | **0.0008** | | **F_(4,64)_ = 6.961** | | **0.0001** | | 10-13 | |
|  | beta (15-30 Hz) | | | **-** | F_(1,16)_ = 1.42 | | 0.2507 | | F_(15,240)_ = 1.66 | | 0.0599 | | - | |
|  | low gamma (31-60 Hz) | | | **-** | F_(1,16)_ = 0.10 | | 0.7604 | | F_(27,432)_ =0.52 | | 0.9785 | | - | |
|  | high gamma (61-90 Hz) | | | **↓** | **F_(1,16)_ = 11.28** | | **0.0040** | | F_(29,464)_ = 0.47 | | 0.9922 | | 61-62 | |

*P* and *F* values are indicated for the effect of the treatment (100 mg/kg modafinil or vehicle) and the interaction between the two factors. *P* and *F* values of significant ANOVAs are shown in bold. Bonferroni *post hoc* comparison test was used to identify frequencies with significant (*P* < 0.05) differences.

**Table S13 Statistical analysis of the effect of chronic modafinil on the quantitative EEG spectra of NREMS and REMS measured during the first 2h of passive phase in R6/2 mice after 6 weeks of treatment**

| **6 weeks of treatment** | |  |  | | | |  | | | |  | |
| --- | --- | --- | --- | --- | --- | --- | --- | --- | --- | --- | --- | --- |
| **Vigilance state** | **EEG Frequency**  **range** | **Direction of change** | **Results of two-way ANOVA**  **(Treatment)** | | | | **Results of two-way ANOVA**  **(Interaction)** | | | | **Frequency ranges with differences**  **(Hz)** | |
|  |  |  | ***F* value** | | ***P* value** | | ***F* value** | | ***P* value** | |  |  |
| **NREMS – passive phase** | |  |  | |  | |  | |  | |  | |
|  | delta (1-4 Hz) | **↓** | **F_(1,16)_ = 5.41** | **0.0335** | | F_(3,48)_ = 2.46 | | 0.0739 | | 4 | |  |
|  | theta (5-9 Hz) | **↓** | **F_(1,16)_ = 7.66** | **0.0137** | | **F_(4,64)_ = 5.04** | | **0.0014** | | 5-6 | |  |
|  | alpha (10-14 Hz) | **↓** | **F_(1,16)_ = 10.51** | **0.0051** | | **F_(4,64)_ = 6.37** | | **0.0002** | | 10-12 | |  |
|  | beta (15-30 Hz) | **↓** | **F_(1,16)_ = 9.29** | **0.0077** | | **F_(15,240)_ = 7.47** | | **<0.0001** | | 15-19 | |  |
|  | low gamma (31-60 Hz) | **-** | F_(1,16)_ = 1.94 | 0.1830 | | F_(27,432)_ = 0.36 | | 0.9989 | | - | |  |
|  | high gamma (61-90 Hz) | **↓** | **F_(1,16)_ = 4.80** | **0.0436** | | **F_(29,464)_ = 2.07** | | **0.0011** | | 61-62 | |  |
| **REMS – passive phase** | |  | |  | |  | |  | |  | |  |
|  | delta (1-4 Hz) | **↓** | **F_(1,16)_ = 6.44** | **0.0219** | | F_(3,48)_ = 1.55 | | 0.2133 | | 3 | |  |
|  | theta (5-9 Hz) | **-** | F_(1,16)_ = 1.08 | 0.3140 | | F_(4,64)_ = 0.69 | | 0.5998 | | - | |  |
|  | alpha (10-14 Hz) | **-** | **F_(1,16)_ = 6.03** | **0.0259** | | F_(4,64)_ = 0.94 | | 0.4460 | | 10 | |  |
|  | beta (15-30 Hz) | **-** | F_(1,16)_ = 4.47 | 0.0505 | | F_(15,240)_ = 0.69 | | 0.7912 | | - | |  |
|  | low gamma (31-60 Hz) | **-** | F_(1,16)_ = 3.53 | 0.0786 | | F_(27,432)_ =0.95 | | 0.5398 | | - | |  |
|  | high gamma (61-90 Hz) | **↓** | **F_(1,16)_ = 5.65** | **0.0303** | | **F_(29,464)_ = 1.99** | | **0.0019** | | 61-62 | |  |

*P* and *F* values are indicated for the effect of the treatment (100 mg/kg modafinil or vehicle) and the interaction between the two factors. *P* and *F* values of significant ANOVAs are shown in bold. Bonferroni *post hoc* comparison test was used to identify frequencies with significant (*P* < 0.05) differences.
